# Quantitative proteomic analyses of dynamic signalling events in cortical neurons undergoing excitotoxic cell death

**DOI:** 10.1038/s41419-019-1445-0

**Published:** 2019-03-01

**Authors:** Ashfaqul Hoque, Nicholas A. Williamson, S. Sadia Ameen, Giuseppe D. Ciccotosto, M. Iqbal Hossain, Jonathan S. Oakhill, Dominic C. H. Ng, Ching-Seng Ang, Heung-Chin Cheng

**Affiliations:** 1 Department of Biochemistry and Molecular Biology, University of Melbourne, Parkville, VIC 3010 Australia; 2 Cell Signalling Research Laboratories, University of Melbourne, Parkville, VIC 3010 Australia; 3 Bio21 Molecular Science and Biotechnology Institute, University of Melbourne, Parkville, VIC 3010 Australia; 4 Metabolic Signalling Laboratory, St. Vincent’s Institute for Medical Research, University of Melbourne, Fitzroy, VIC 3065 Australia; 5 Department of Pharmacology and Therapeutics, University of Melbourne, Parkville, VIC 3010 Australia; 6 Mary MacKillop Institute for Health Research, Australian Catholic University, Melbourne, Victoria 3000 Australia; 7 School of Biomedical Sciences, University of Queensland, St. Lucia, QLD Australia

## Abstract

Excitotoxicity, caused by overstimulation or dysregulation of ionotropic glutamate receptors (iGluRs), is a pathological process directing neuronal death in many neurological disorders. The aberrantly stimulated iGluRs direct massive influx of calcium ions into the affected neurons, leading to changes in expression and phosphorylation of specific proteins to modulate their functions and direct their participation in the signalling pathways that induce excitotoxic neuronal death. To define these pathways, we used quantitative proteomic approaches to identify these neuronal proteins (referred to as the changed proteins) and determine how their expression and/or phosphorylation dynamically changed in association with excitotoxic cell death. Our data, available in ProteomeXchange with identifier PXD008353, identified over 100 changed proteins exhibiting significant alterations in abundance and/or phosphorylation levels at different time points (5–240 min) in neurons after glutamate overstimulation. Bioinformatic analyses predicted that many of them are components of signalling networks directing defective neuronal morphology and functions. Among them, the well-known neuronal survival regulators including mitogen-activated protein kinases Erk1/2, glycogen synthase kinase 3 (GSK3) and microtubule-associated protein (Tau), were selected for validation by biochemical approaches, which confirmed the findings of the proteomic analysis. Bioinformatic analysis predicted Protein Kinase B (Akt), c-Jun kinase (JNK), cyclin-dependent protein kinase 5 (Cdk5), MAP kinase kinase (MEK), Casein kinase 2 (CK2), Rho-activated protein kinase (Rock) and Serum/glucocorticoid-regulated kinase 1 (SGK1) as the potential upstream kinases phosphorylating some of the changed proteins. Further biochemical investigation confirmed the predictions of sustained changes of the activation states of neuronal Akt and CK2 in excitotoxicity. Thus, future investigation to define the signalling pathways directing the dynamic alterations in abundance and phosphorylation of the identified changed neuronal proteins will help elucidate the molecular mechanism of neuronal death in excitotoxicity.

## Introduction

Excitotoxicity is a prominent pathological process directing neuronal death. It is initiated by aberrant stimulation of neurons by the excitatory neurotransmitter glutamate, which overactivates the ionotropic glutamate receptors (iGluRs) including *N*-methyl-d-aspartate (NMDA) receptor, 2-amino-3-(5-methyl-3-oxo-1,2-oxazol-4-yl) propanoic acid (AMPA) receptor and kainate receptor^1^. Under physiological conditions, these receptors maintain neural development and survival as well as regulate synaptic plasticity^2^. However, in neuropathological conditions such as cerebral ischaemia, traumatic brain injury and Zika virus-induced brain damage^2–5^, overstimulation and/or dysregulation of these receptors lead to neuronal death^6–9^. Mechanistically, the overstimulated receptors allow excessive influx of Ca^2+^ into cytosol of the affected neurons, leading to the aberrant activation of specific proteases, phospholipases and endonucleases as well as initiation of excessive production of nitric oxide, arachidonic acid metabolites and reactive oxygen species to cause neuronal death^10–13^. The exact mechanism by which these cellular events contribute to neuronal death remains unclear. Deciphering this mechanism entails identification of the key signalling cellular events triggered by aberrant stimulation of the iGluRs as well as elucidation of how they interplay both spatially and temporally to direct neuronal cell death. Calcium-dependent protease calpain, nitric oxide synthase and NADPH oxidase 2 are overactivated by the excessive cytosolic calcium during the early stage of excitotoxicity^14–16^. Their overactivation perturbs the expression and/or phosphorylation of specific neuronal proteins to direct cell death^14,15,17,18^. Our ultimate goal is to decipher the signalling pathways directing excitotoxic neuronal death. To this end, we employed the stable isotope dimethyl labelling-based quantitative proteomic approaches to identify proteins exhibiting significant changes in abundance and/or phosphorylation (referred to as changed neuronal proteins) in cultured primary cortical neurons, in response to glutamate overstimulation. With this approach, we identified multiple changed neuronal proteins at different time points following glutamate treatment. Among them, we chose three well known neuronal survival regulators including mitogen-activated protein kinases Erk1/2, glycogen synthase kinase 3 (GSK3) and the microtubule-associated protein Tau (also referred to as Mapt) for validation by biochemical approaches, which confirmed our proteomic findings. We then adopted a standard bioinformatic procedure to predict the identities and dynamics of changes of activation states of the potential upstream protein kinases phosphorylating some of the changed neuronal proteins, as well as their involvement in excitotoxic neuronal death. To examine the predictive accuracy of this bioinformatic procedure and the dataset generated from our proteomic study, we selected two predicted upstream protein kinases Akt and CK2 for further biochemical analyses. Results of the analyses confirm the predictions. For most of the changed neuronal proteins and their upstream protein kinases we identified, this is the first report documenting their involvement in excitotoxicity. Thus, our findings can form a conceptual framework for future investigation to chart the signalling pathways directing neuronal death in excitotoxicity.

## Results

### Cultured primary cortical neurons exhibited significant reduction in cell survival, morphological changes and characteristics of necrosis after treatment with excess glutamate

**Fig. 1:**
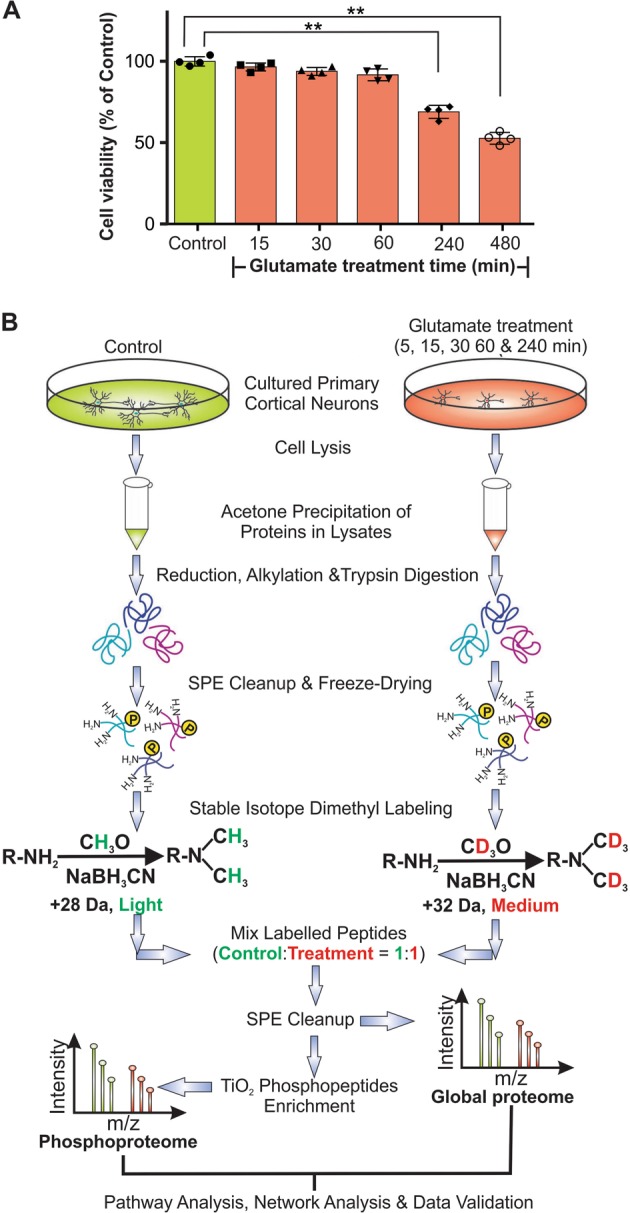
Workflow to quantitatively measure the temporal changes of global and phospho-proteomes of neurons induced by glutamate treatment. **a** Cultured cortical neurons at DIV7 (cell number: ~6 × 10^5^) were treated with 100 μM of glutamate. MTT cell viability assay showing the time-dependent changes in cell viability of cultured neurons in response to glutamate treatment (orange bars) and control (green bar) untreated neurons. The viability of the glutamate-treated neurons compared with that of the control neurons is presented as mean ± SD (*n* = 4, ** indicates *p* < 0.01, one-way ANOVA with Dunnett’s multiple comparison test). The morphological changes of the neurons at 5, 15, 30, 60 and 240 min after glutamate treatment are presented in Figures S1 and S2. **b** Workflow of proteomic analysis of cultured primary cortical neurons treated with glutamate for 5, 15, 30, 60 and 240 min. Cell lysis was performed on glutamate treated and untreated (control) neurons using RIPA buffer. Total proteins from these neuronal lysates were precipitated with acetone followed by reduction, alkylation and digestion with trypsin. The resultant tryptic peptides were purified by reverse-phase solid phase extraction cartridges (SPE) and freeze-dried prior to stable isotope dimethyl labelling with formaldehyde (CH_2_O, light label, green) for peptides derived from the untreated neurons and deuterated formaldehyde (CD_2_O, medium label, red) for peptides derived from the treated neurons in the presence of sodium cyanoborohydride (NaBH_3_CN). Around 200 μg of proteins from neuronal lysates were used as starting material. After tryptic digestion and differentiated isotopic dimethyl labelling, equal amounts of the derivatized peptides from proteins in the lysates of both treated and untreated neurons were mixed (i.e., control: treatment, 1:1). Aliquots of 50 μg of mixed labelled tryptic peptides were used for global proteomic analysis. An aliquot of mixed labelled peptides was set aside and later injected to LTQ Orbitrap Elite mass spectrometer for LC-MS/MS analysis. The data obtained (global proteome) reveals the temporal global proteome changes of neurons induced by glutamate treatment. The remaining portions of the mixed labelled peptides were enriched for phosphopeptides using TiO_2_ micro-columns prior to injection to LTQ Orbitrap Elite mass spectrometer for LC-MS/MS analysis. The data obtained (phosphoproteome) reveals the temporal phosphoproteome changes of neurons induced by glutamate treatment. *n = 3 for all time points from 5 min to 60 min and *n* = 6 for 240 min

Mouse primary cortical neurons after 7 days of culturing were used as the model systems because they have morphologically developed axons, dendrites and synaptic boutons (Figures S1-S2) as well as expressing all the NMDA receptor subunits^18^. To mimic the excitotoxic condition that leads to neuronal death in acute neurological disorders, the neuronal cultures were treated with glutamate at the neurotoxic concentration (100 μM)^18^ and the biochemical changes were investigated by proteomic analysis. While no significant change in cell viability was found at the early treatment time period from 15 to 60 min, a significant reduction in cell viability was evident at 240 and 480 min (Fig. 1a). Furthermore, significant reduction in the density of dendrites and axons, and biochemical features of necrosis were noticeable at 240 min of glutamate treatment (Figures S1-S3 and Supplemental Results).

Our ultimate goal was to identify the key cellular events mediating the excitotoxic signals originating from the overstimulated iGluRs. Since these events occur at the initial phase of the cytotoxic signalling pathway, their identification is best conducted with glutamate-treated neurons showing no signs of cellular damage but would subsequently progress to excitotoxic cell death. With no signs of reduced viability from 15 to 60 min of glutamate treatment (Fig. 1a), we selected time points from 5 to 60 min to investigate how the abundance and phosphorylation of neuronal proteins are perturbed at the early stage of excitotoxicity (Fig. 1b). Additionally, we also aimed to identify the cellular events associated with neuronal loss. To this end, we chose neurons at 240 min after glutamate treatment for analysis because they exhibited significantly reduced viability, altered morphological features and biochemical features of necrosis (Fig. 1a, S2-S3). We predicted that quantitative analysis of the changes in both global and phospho-proteomes of neurons at 5–240 min after glutamate treatment could unveil the cellular events occurring at early to late stages of excitotoxicity. Some of these events, initiated in the early stage of excitotoxicity when neurons are still alive, are potential driver events directing excitotoxic cell death^8,19^.

### Global proteomic analysis revealed changes in the abundance of multiple neuronal proteins after glutamate overstimulation for up to 240 min

Following stable isotope dimethyl labelling of tryptic digests of proteins from neuronal lysates, all samples were subjected to LC-MS/MS analysis to monitor changes in the global proteome by following the workflow depicted in Fig. 1b. A total of 1250 neuronal protein groups were identified on a targeted 1% false discovery rate (FDR) at the peptide and protein levels (Table S1). More than 95% of these proteins were identified with 2 or more unique peptides. The relative abundance of all protein molecules identified in at least two biological replicates for each of the treatment time points are presented in a heatmap (Figure S4), which shows time-dependent changes in abundance of many proteins. The correlations between replicates of the proteomic changes at different time points are depicted in a multi-scatter plot (Figure S5).

**Fig. 2:**
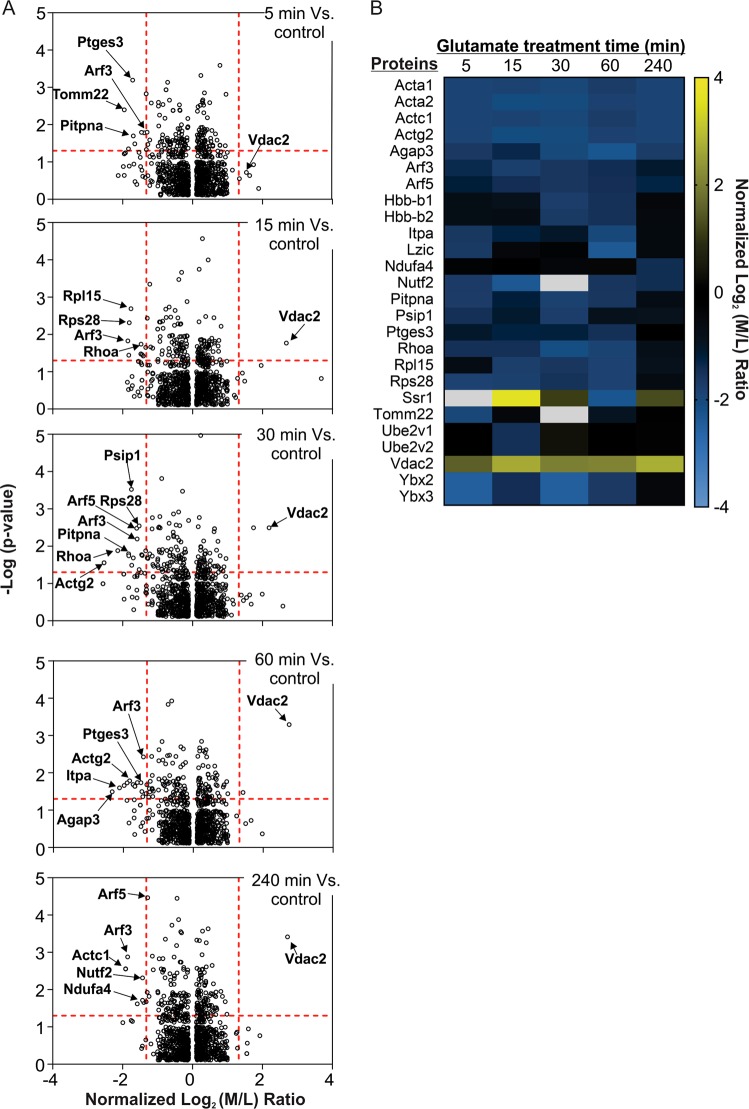
Neuronal proteins exhibiting significant temporal changes in abundance in response to glutamate overstimulation. **a** Volcano plots depicting the distribution of quantified neuronal proteins identified at 5, 15, 30, 60 and 240 min of glutamate treatment. Selected neuronal proteins showing significant changes (±2.5-fold changes, *p* ≤ 0.05) in abundance are also highlighted **b** Heatmap depicting the time-dependent changes in abundance of selected neuronal proteins in response to glutamate treatment. Only proteins exhibiting ± 2.5-fold differences in abundance in at least one of the five time points are selected for presentation. The temporal changes (abundance ratios) of all identified proteins are presented in Table S1. White boxes indicate that the abundance ratio information is missing for those given proteins in any of the replicate samples

Of the identified differentially expressed protein molecules, at least 26 neuronal proteins showed a 2.5-fold change in abundance following glutamate treatment in at least one of the time points examined. Changes of the abundance of these proteins are presented in a heatmap (Fig. 2b). Some of these selected proteins are highlighted in the volcano plots derived from global proteome data (Fig. 2a). With the exception of Vdac2 and Ssr1, Fig. 2b reveals that most identified changed protein exhibited reduced abundance following glutamate overstimulation. Presumably, their reduced abundance was caused by proteolytic degradation by neuronal proteases such as cathepsins, calpains and those in ubiquitin proteasome system activated in excitotoxicity^20,21^.

### Phosphoproteomic analysis revealed dynamic changes in phosphorylation levels of multiple neuronal proteins induced by glutamate overstimulation

**Fig. 3:**
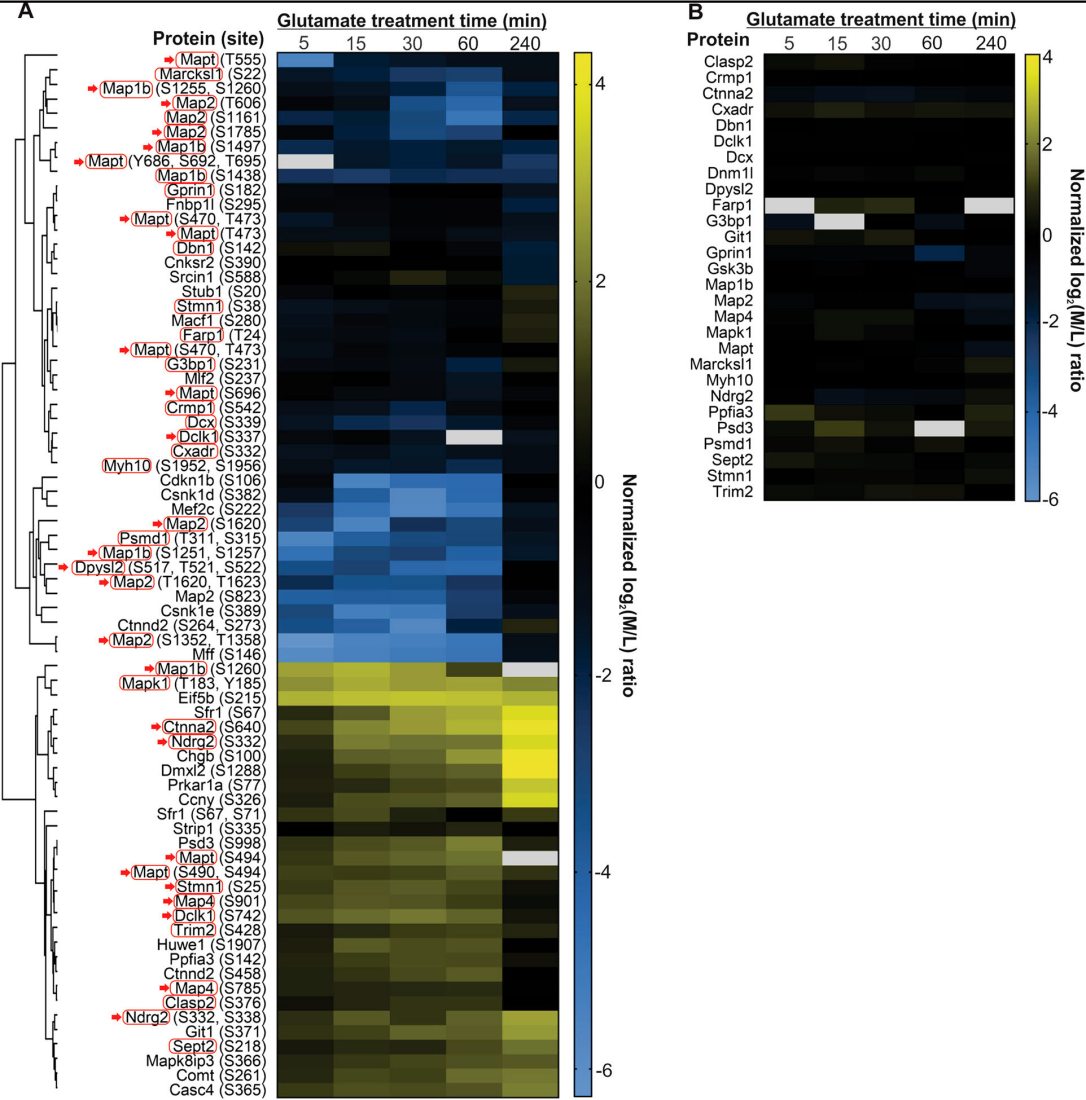
Temporal changes in phosphorylation states of neuronal proteins in response to glutamate overstimulation. **a** Heatmap depicting the time-dependent changes of phosphorylation levels of specific phosphosites in selected neuronal proteins in response to glutamate treatment. Phosphoproteins of which the relative protein level abundance information are available from global proteome data are labelled with red box. Phosphoproteins with more than one identified phosphosite are marked with red arrows. Phosphosites exhibiting similar patterns of temporal changes are grouped in clusters. **b** Heatmap depicting the changes in abundance of the neuronal proteins depicted in panel **a**. White boxes indicate that the abundance ratio information is missing for those given proteins in any of the replicate samples obtained at that selected time points

Aliquots of the mixed labelled tryptic digests derived from all five time points of glutamate treated neuronal lysates were enriched for phosphopeptides using TiO_2_ microcolumns prior to LC-MS/MS analysis (Fig. 1b). Our procedures identified a total of 972 phosphopeptide group combinations derived from 383 different neuronal proteins. Some of the phosphopeptides contain more than one phosphorylation site, suggesting that they are the targets of multiple protein kinases and/or phosphatases (Fig. 3 and Table 1, Table S2 and S3). Correlations between replicates of phosphoproteomic changes at different time points are depicted in a multi-scatter plot (Figure S6). One sample t-test was utilized to identify the neuronal proteins of which the phosphorylation was significantly perturbed by the glutamate treatment over time (Table S2). Changes of the phosphopeptides showing a 2.5-fold increase or decrease in their phosphorylation levels in at least one of the five examined time points following the glutamate treatment are presented in a heatmap (Fig. 3a). The identified phosphosites and the corresponding phosphoproteins are also presented in volcano plots (Figure S7). Changes in the abundance of the corresponding identified phosphoproteins were retrieved from the global proteome data and presented in Fig. 3b. At least 72 phosphosites from 50 neuronal proteins were found to undergo significant changes in phosphorylation levels. Among these phosphosites, some exhibited a time-dependent increase in their abundance, whereas the majority of sites exhibited decrease in abundance that occurred independently of protein degradation (Fig. 3b).

**Table 1:** Selected neuronal proteins with at least one site exhibited increased phosphorylation in response to glutamate overstimulation




Prediction of the upstream kinase(s) targeting the identified neuronal proteins either activated or with at least one phosphosite exhibiting increased phosphorylation in response to glutamate treatment. Based upon the western blot data shown in Fig. 7 that revealed reduced phosphorylation of S21 of GSK3α, the phosphopeptide consisting of S21 derived from GSK3α is listed. For proteins such as Mapt (Tau) and Map1b containing multiple phosphorylation sites, the changes in phosphorylation states of other identified sites are presented. For NetworKIN prediction of upstream kinases, minimum score and maximum difference were set at 2 and 4, respectively

The coloured letters highlight the identified phosphorylated amino acid residues

Although our study revealed for the first time many neuronal proteins exhibiting changes in phosphorylation levels in excitotoxicity, we failed to detect changes in the phosphorylation states of several well-known mediators of neuronal death such as p53, p73 and PTEN^22–25^. Since the coverage of phosphoproteome of the label-free quantitative phosphoproteomic method^26^ is better than those of other methods, its use in future investigations of excitotoxicity research will uncover more changed neuronal proteins.

### The identified changed neuronal proteins are predicted to contribute to defective neuronal functions and morphology

For the changed neuronal proteins exhibiting significant perturbations (±2.5-fold changes) in abundance and phosphorylation in response to glutamate treatment, Ingenuity Pathway Analysis (IPA) software revealed that many of them are components of the canonical signalling pathways and cellular processes that regulate cell morphology, actin cytoskeleton signalling, and cell–cell and cell-extracellular matrix communications. Furthermore, their perturbed expression and/or phosphorylation are related to brain damage associated with neurodegenerative diseases and acute neurological disorders (Figure S8). IPA also predicted the interaction networks for some of the changed proteins. The top three ranked networks suggest that some of the identified changed neuronal proteins are functionally linked to the protein kinases Erk1/2, the microtubule associated protein Tau and neurotrophic receptor tyrosine kinase 1 (NTRK1) known to govern neuronal survival (Figure S9)^27–31^.

### Biochemical analyses validated the changes in the phosphorylation states of Erk1/2 and Tau in excitotoxicity revealed by proteomic analyses

**Fig. 4:**
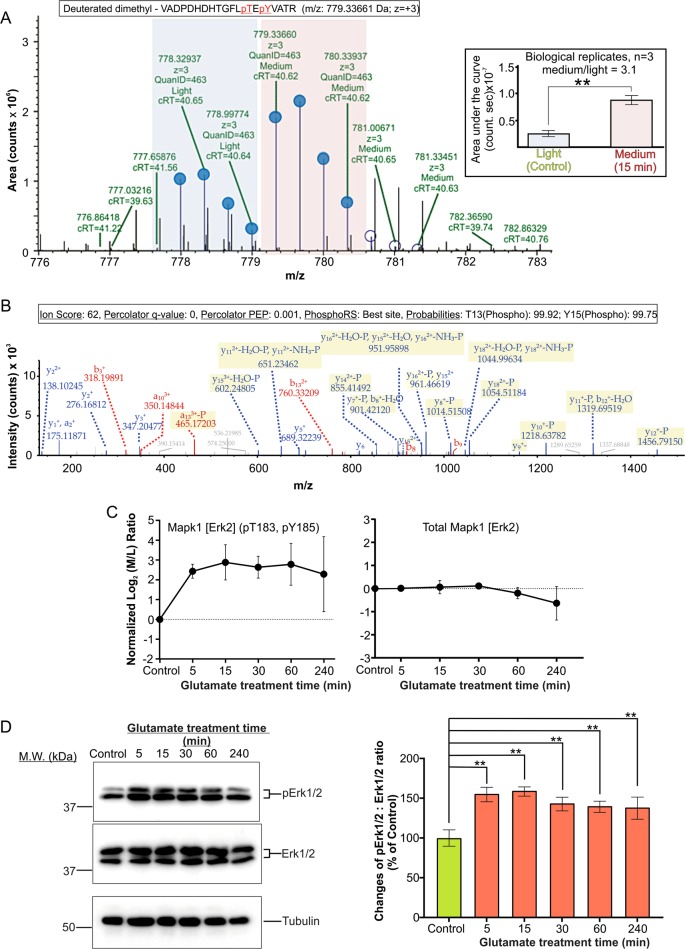
Validation of the proteomic result indicating activation of neuronal Erk1/2 by glutamate overstimulation. **a** Extracted ion chromatogram (XIC) showing isotopic clusters used for MS1 quantification of the dimethyl- (light, blue shaded region) and deuterated dimethyl-labelled (medium, pink shaded region) tryptic phosphopeptides derived from phosphorylated Erk2 extracted from the control and 15 min glutamate-treated neurons. The calculated average median value of the medium: light ratios from three biological replicates = 3.1 (data presented as mean of the area under the curves ± SD, **p < 0.01) **b** The MS2 spectrum used for identification of this unique phosphopeptide with a Mascot ion score of 62. **c** Plots of the time-dependent changes in abundance of total Mapk1 (also referred to as Erk2) and the abundance of the tryptic phosphopeptide consisting phospho-T183 and phospho-Y185 derived from Mapk1 (Erk2). The ratios of the peptides derived from Mapk1 (Erk2) of the treated neurons relative to those of the control neurons are presented as the mean of normalised log_2_ medium/light ratios at different time points following glutamate treatment. Data presented as mean ± SD, *n* = 3 for the 5–60 min time points and *n* = 6 for the 240 min time point. **d** Left panel western blot analysis of the phosphorylation state of Mapk3/1 (Erk1/2). Right panel: Changes of the ratio of the densitometric units of the signal of phospho-Erk1/2 (pErk1/2) versus those of the signal of total Erk1/2. A value of 100% is assigned to the pErk1/2:Erk1/2 ratio of the control neurons. Data presented as mean ± SD, *n* = 3 and ** indicates *p* < 0.01, one-way ANOVA with Dunnett’s multiple comparison test

We chose Erk1/2 and Tau for biochemical studies to validate our proteomic findings because they were the predicted hubs of interactions with many identified changed neuronal proteins in excitotoxicity (Figure S9). The phosphoproteomic data presented in Fig. 4a–c, Table 1, Figure S4 show that the phosphorylation level of Erk2 (also referred to as Mapk1) was increased after glutamate treatment by three-fold at 5 min and the increase remained until 60 min with a slight reduction at 240 min. The phosphosites in the identified phosphopeptide correspond to the conserved threonine and tyrosine residues critical for activation of Erk1 and Erk2 (Thr-183 and Tyr-185 for Erk2 and Thr-202 and Tyr-204 for Erk1)^32,33^.

The mass spectra (Fig. 4a, b) show that the dimethyl labelled phosphopeptide of Erk2 from neurons treated with glutamate for 15 min is significantly more abundant than that from the untreated neurons. Examination of the mass spectra of the phosphopeptides of the control versus glutamate-treated neurons at other time points also reveals a significant increase in its abundance in the glutamate-treated neurons. However, the increased abundance of this phosphopeptide in glutamate-treated neurons was not accompanied by any significant change in the other unique peptides derived from Erk2 (Mapk1) in the global proteome data, indicating that the increased abundance of the phosphopeptide is resulted from the increased phosphorylation stoichiometry of Erk2 at Thr-183 and Tyr-185.

Both Erk1 and Erk2 are activated in parallel by phosphorylation of these conserved threonine and tyrosine residues by their upstream regulatory kinase MEK^32,33^. Hence, the increase in phosphorylation levels of Thr-183 and Tyr-185 of Erk2 suggests increased phosphorylation of Erk1 (Mapk2) at Thr-202 and Tyr-204 in excitotoxicity. Our western blotting results validate the proteomic findings—glutamate treatment induced a rapid and sustained increase in phosphorylation of both neuronal Erk1 and Erk2 at the two conserved residues (Fig. 4d).

**Fig. 5:**
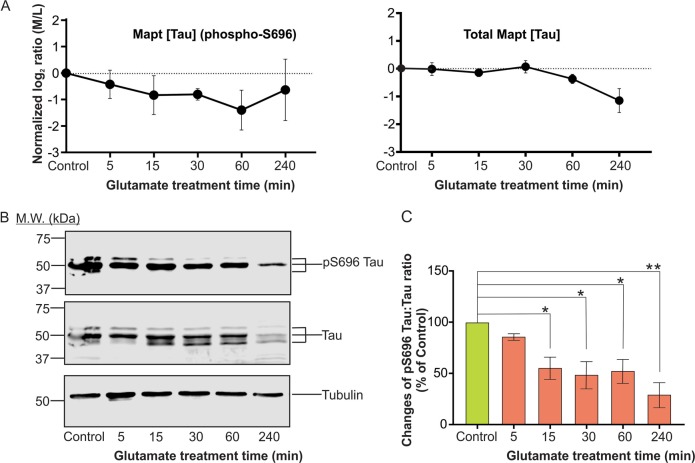
Validation of the proteomic data showing time-dependent changes in the abundance and the level of phosphorylation at Ser-696 of Mapt (Tau). **a** The changes in abundance of the tryptic phosphopeptide consisting of phosphorylated Ser-696 (derived from phosphoproteome data) and the unique tryptic peptide(s) used for the identification of total Tau following glutamate treatment at different time points. The abundance of the tryptic peptides is presented as mean of the normalized log_2_ ratios of the medium labelled versus light labelled (M/L) dimethyl derivatized tryptic peptides, error bars represent standard deviations. **b** Western blot analysis to follow the total Tau and phospho-Ser-696 level of control neurons and neurons at varying time points after glutamate overstimulation. **c** Ratios of the densitometric units of the signals of phospho-Tau at Ser-696 versus those of the signal of total Tau in the control and glutamate-treated neurons were calculated. The changes in the ratio of the glutamate-treated neurons in comparison with that of control are presented. Data presented as mean ± SD, *n* = 3, ** indicates *p* < 0.01 and * indicates *p* < 0.05, one-way ANOVA with Dunnett’s multiple comparison test

We identified multiple phosphosites in neuronal Tau (Uniprot ID: P10637-1; Mapt), which exhibited time-dependent changes in phosphorylation level in excitotoxicity. The identified sites and the homologous residues in human Tau (Uniprot ID: P10636-8; in bracket and with “h” in the superscript) are: Ser-470 (A178^h^), Thr-473 (T181^h^), Ser-490 (S198^h^), Ser-494 (S202^h^), Thr-555 (T263^h^), Ser-696 (S404^h^), Thr-678 (T386^h^), Ser-692(S399^h^), Thr-695(T403^h^) and Tyr-686 (Y394^h^). In response to glutamate overstimulation, Ser-696 (S404^h^) phosphorylation level was significantly reduced (Fig. 5a). Besides inducing changes in phosphorylation states, glutamate overstimulation also caused significant reduction in the expression level of Tau (at least at 240 min) as revealed by data of global proteomics analysis (Figs. 3b and 5a). To validate these findings, we performed western blot analysis to monitor the expression level and phosphorylation state of Ser-696 of neuronal Tau at varying time points after glutamate overstimulation. Phospho-Ser-696 was chosen because specificity of the anti-pS696 Tau antibody has been confirmed^28,29^. In agreement with our proteomic data, western blot analysis revealed significant reduction in the expression of Tau and its phosphorylation at Ser-696 induced by glutamate overstimulation (Fig. 5b, c).

### GSK3, Rock, Cdk5, JNK, MEK, SGK1 and CK2 are potential upstream protein kinases targeting some of the changed neuronal proteins in excitotoxicity

The interaction networks (Figure S9) suggest potential direct and indirect interactions among subsets of the changed neuronal proteins in excitotoxicity. However, they do not predict the upstream regulatory enzymes such as protein kinases and phosphatases directing the changes in expression and phosphorylation states of these selected neuronal proteins. Furthermore, they do not predict how changes in phosphorylation levels of the identified neuronal proteins affect their enzymatic activities and/or biological functions. We therefore conducted more in-depth bioinformatic and literature searching-based analyses to chart the signalling pathways governing the changes in expression and phosphorylation states of the neuronal proteins listed in Figs. 2 and 3.

**Fig. 6:**
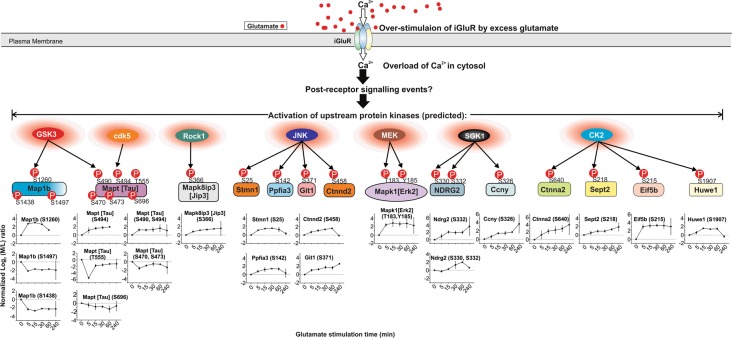
Predicted protein kinases contributing to the temporal changes of phosphorylation states of selected proteins in neurons undergoing excitotoxic cell death. Temporal changes of the phosphorylation states of neuronal Map1b, Tau, Jip3, Git1, Stmn1, Ppfia3, Erk1/2, Ndrg2, cyclin Y, Eif5b, Ctnna2 and Ctnnd2 at 5, 15, 30, 60 and 240 min after glutamate overstimulation. These proteins have at least one phosphosite exhibiting time-dependent increase in phosphorylation level. The upstream protein kinases targeting these sites are predicted by comparing the phosphosite sequences with the known optimal phosphorylation sequences of protein kinases and literature-curated knowledge. These predicted upstream kinases including GSK3, cdk5, Rock1, JNK, MEK, SGK1 and CK2 are activated by specific post-receptor signalling events initiated by overstimulation of the neuronal iGluRs. The question mark indicates that the post-receptor signalling events directing activation of the predicted upstream kinases remain unclear. All data shown in the plots are the mean of normalised log_2_ medium/light ratios at different time points following glutamate treatment. The error bars denote standard deviations. The plot depicting the time-dependent changes of the phosphorylation of Mapt (Tau) at Ser-696 in excitotoxicity is also shown in Fig. 5a

An approach to chart the signalling pathways in excitotoxicity is to define the upstream protein kinases and phosphatases targeting the identified neuronal proteins, which exhibited changes in phosphorylation states in excitotoxicity. A number of the identified phosphosites exhibited a significant time-dependent increase in phosphorylation level in at least one of the time points after glutamate treatment without significant changes in their abundance (Fig. 3). To predict the upstream kinases phosphorylating these sites, we adopted a bioinformatic analysis procedure that constitutes two steps. In Step 1, sequences of the selected phosphosites are compared with the consensus optimal phosphorylation sequences of known protein kinases^34^. In Step 2, a literature search was performed to examine if the selected phosphosites were previously found to be phosphorylated by the predicted kinases in neurons and/or other cell types. Results of the searches are recorded in Table 1. To complement the results obtained from this procedure, we employ the NetworKIN algorithm to predict the upstream kinases of the selected identified phosphosites^35^. Both methods predicted glycogen synthase kinase 3α- and β-isoforms (GSK3α/β), Rho-activated protein kinase (Rock), cyclin-dependent protein kinase 5 (Cdk5), c-Jun protein kinase (JNK), mitogen-activated protein kinase kinase (MEK), serum/glucocorticoid regulated kinase-1 (SGK1) and Casein Kinase 2 (CK2) as the upstream kinases targeting some of these phosphosites exhibiting increased phosphorylation levels (Fig. 6 and Table 1). The temporal changes in phosphorylation levels of the phosphosites presented in Fig. 6 reflect the dynamics of changes of the activation states of these kinases in excitotoxicity. We hypothesize that some of these predicted kinases, upon activation, mediate the neurotoxic signals emanating from the overstimulated glutamate receptors. Among them, aberrant activation of GSK3β, Cdk5, MEK and JNK are known to contribute to excitotoxic neuronal death^36–39^. How neuronal Rock, SGK1 and CK2 undergo changes in activation state is poorly understood. The predicted rapid and sustained activation of them in excitotoxicity justifies further investigations to examine how their activities are regulated in excitotoxicity by biochemical approaches.

### Neuronal Akt and GSK3α/β underwent dynamic changes in activation state in excitotoxicity

**Fig. 7:**
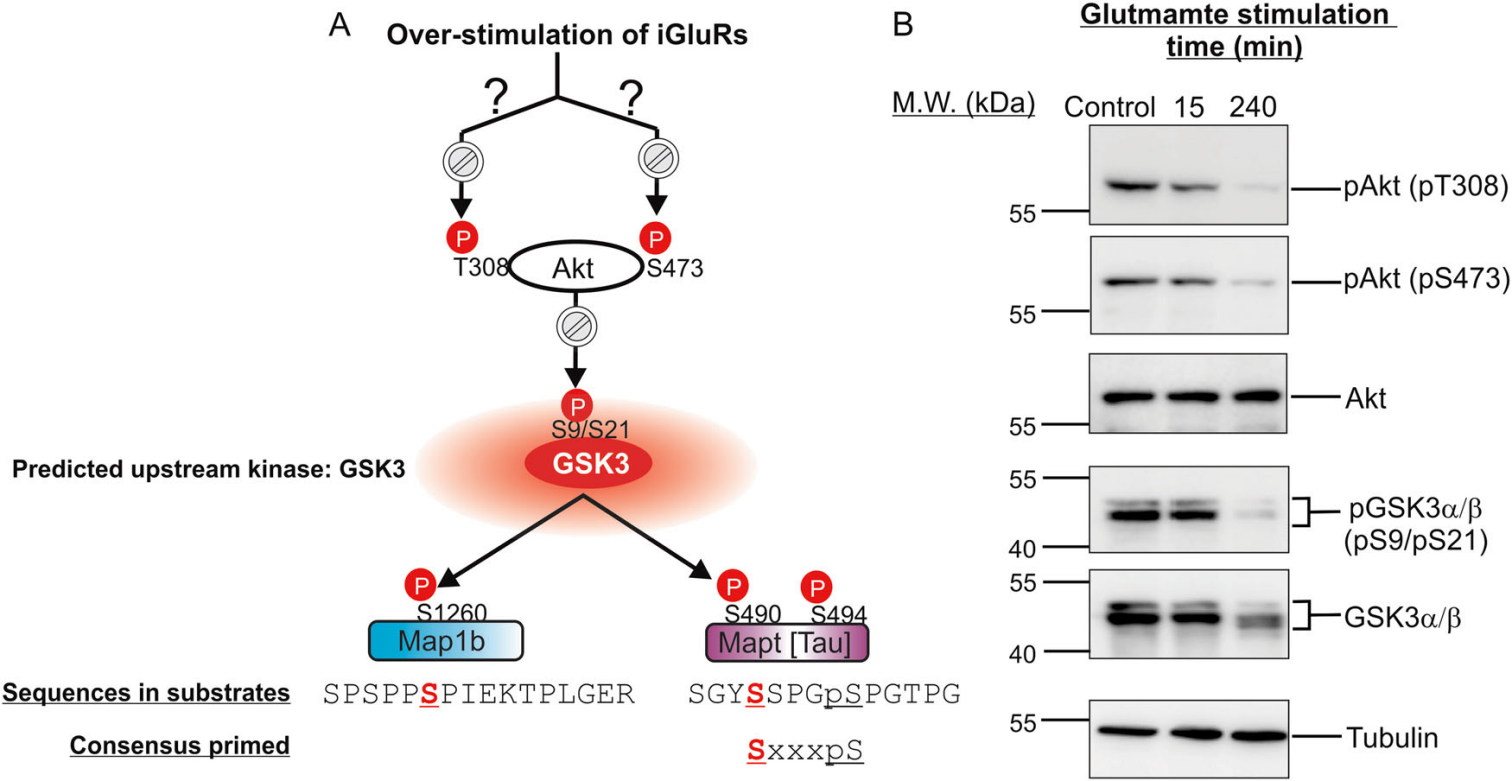
Confirmation of the perturbation of GSK3 and Akt signalling activities in excitotoxicity predicted by changes of the phosphorylation states of Map1b and Mapt (Tau). **a** A model depicting how glutamate overstimulation leads to inactivation of Akt and activation of GSK3 in neurons in excitotoxicity (top panel). GSK3 was previously found to phosphorylate Ser-1260 of Map1b and Ser-490 of Tau in vitro and in cells. GSK3 phosphorylation of Ser-490 of Tau is dependent on prior phosphorylation of Ser-494 by Cdk5. The sequences around Ser-1260 of Map1b and Ser-490 of Tau as well as the consensus phosphorylation-primed optimal sequences of GSK3 protein substrates are shown. The plots showing changes in phosphorylation states at Ser-1260 of Map1b and Ser-490/Ser-494 of Tau, presented as normalized log_2_ ratios of medium-versus light-labelled dimethyl derivatized tryptic phosphopeptides, are shown in Fig. 6. **b** Western blot analysis to follow the changes of phosphorylation states of the activating phosphorylation sites (Thr-308 and Ser-473) of Akt, and the inhibitory phosphorylation site (Ser-21 and Ser-9 of GSK3α and GSK3β) of the two GSK3 isoforms at 15 and 240 min after glutamate overstimulation. Besides the western blots probed with the phospho-specific antibodies, the western blots of total Akt, total GSK3α/β and tubulin are also presented

GSK3α/β recognize the motif SxxxpS in protein substrates as the consensus optimal phosphorylation sequences (Fig. 7a)^40^. The SxxxpS motif, where the underlined S stands for the phosphorylation site, pS stands for phospho-serine and x stands for any amino acid residue, is referred to as the consensus primed phosphorylation sequence. Phosphorylation of this motif is dependent on the presence of pS three residues away at the C-terminal side of the phosphorylation site. Thus, phosphorylation of the target serine residue (S) is “primed” by prior phosphorylation of the C-terminal serine residue by another protein kinase. Ser-490 of Tau, locating in a motif that conforms with the SxxxpS sequence (Fig. 7), is a potential GSK3α/β phosphorylation site. This prediction is supported by a previous report of phosphorylation of Ser-490 of Tau by GSK3α/β^41^. Besides the consensus primed phosphorylation sequence, GSKα/β also preferentially phosphorylate a small subset of their protein substrates at specific serine and threonine residues without a nearby primed phosphorylated residue;^42^ they are referred to as non-primed GSK3 substrates^40^. Map1b is one of the non-primed GSK3 substrates as its Ser-1260 is phosphorylated by GSK3 without prior phosphorylation of a nearby residue in neurons^42^. Our phosphoproteomic analysis revealed time-dependent increase in abundance of the tryptic phosphopeptide consisting of phospho-Ser-1260 of Map1b and that of phospho-Ser-490 and phospho-Ser-494 of Tau in neurons from 5 to 240 min after glutamate overstimulation (Figs. 6 and 7). Based on these experimental and analytic results, we predict that neuronal GSK3α/β were activated as early as 5 min after glutamate treatment and the activation was sustained till 240 min post treatment.

**Fig. 8:**
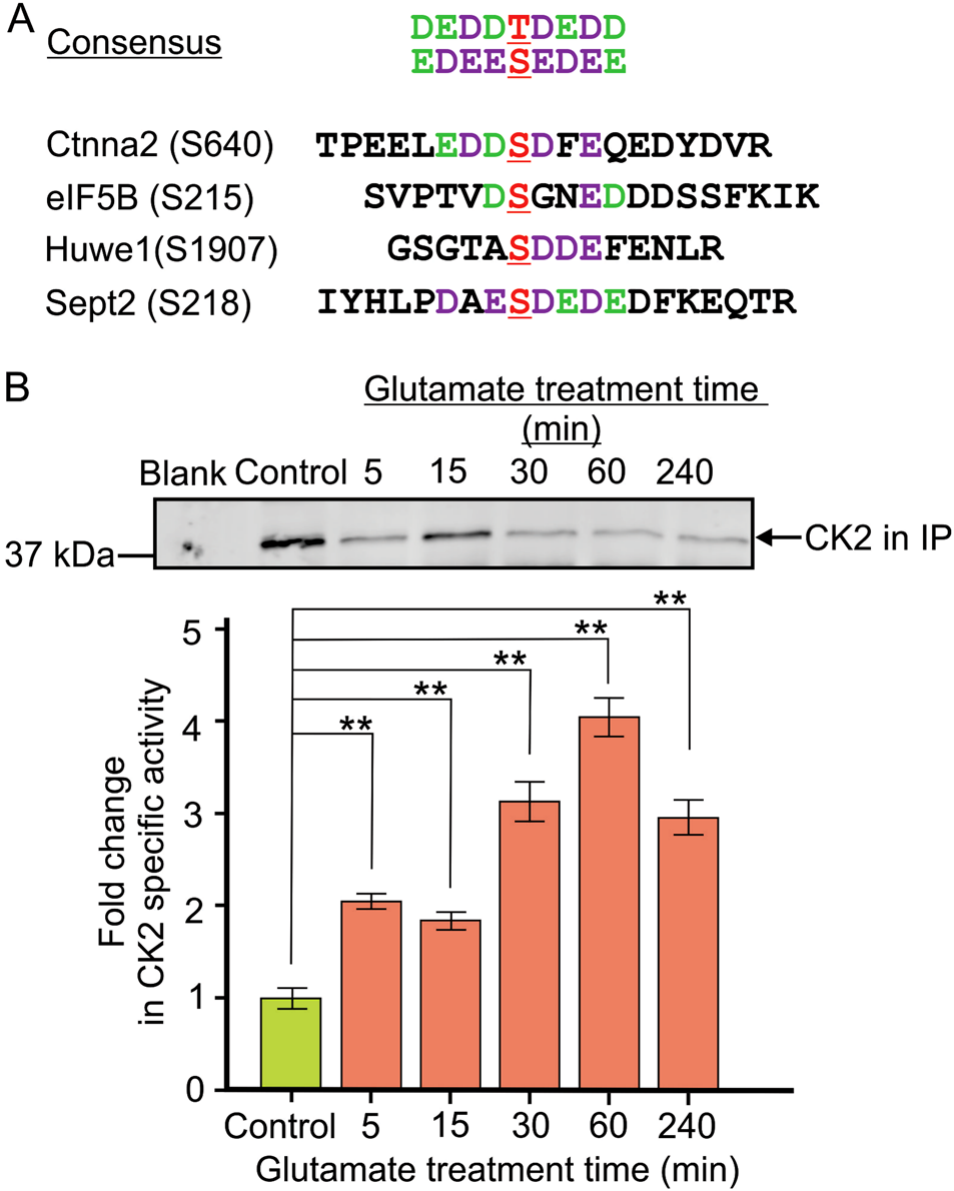
Confirmation of the activation of neuronal CK2 in excitotoxicity predicted by the changes of phosphorylation states of Ctnna2, eIF5B, Huwe1 and Sept2. **a** Sequences of the perturbed phosphosites in Ctnna2, eIF5B, Huwe1 and Sept2 show significant conformity to the consensus phosphorylation sequence of CK2 defined by the peptide library approaches^45^. The phosphorylation sites (S and T) are in red fonts and underlined. The preferred residues in each position of the consensus CK2 phosphorylation sequence are in purple and green fonts. The peptide library approach revealed that CK2 exhibited a higher preference for residues labelled in purple than those labelled in green^45^. **b** Upper panel: Part of the western blot showing the amounts of CK2 immunoprecipitated from lysates of control and glutamate treated neurons for determination of CK2 specific kinase activity. The image of the whole blot is shown in Figure S10. The amounts of CK2 in the immunoprecipitates were measured by densitometric analysis of the anti-CK2 signals of the western blot. Lower panel: comparison of the specific activity of CK2 in untreated neurons (control) and neurons after 5–240 min after glutamate overstimulation. The kinase activity of the immunoprecipitated CK2 was measured by the efficiency of its phosphorylation of the CK2-specific peptide substrate CK2-tide modelled after the consensus phosphorylation sequence of CK2 (Fig. 8a). The specific activity of the immunoprecipitated CK2 was expressed as the rate of phosphorylation of the CK2-tide per densitometric unit of the anti-CK2 immunoreactive signal. The results are presented as the ratios of the specific kinase activity of immunoprecipitated CK2 of the glutamate-treated neurons versus those of the untreated (control) neurons. Data presented as a fold-change increase in specific CK2 activity in control and treated neuronal lysates. Data are presented as mean ± SD; *n* = 3; ** indicates *p* < 0.01, one-way ANOVA with Dunnett’s multiple comparison test

GSK3α/β kinase activity is suppressed by phosphorylation by Akt at a conserved serine residue (Ser-9 of GSK3β and Ser-21 of GSK3α)^43^. Of relevance, we identified a long singly phosphorylated peptide (^17^T…K^50^; Percolator *Q* value = 0, PEP = 5.07e^−6^) containing multiple Ser and Thr residues derived from GSK3α, where the search engine and ptmRS algorithm was unable to pinpoint the exact phosphorylation site (Table S2). This phosphopeptide, containing Ser-21, exhibits decreased abundance in several treatment points (Table 1 and S2). These results support our prediction that GSK3α and β are activated as a result of reduced phosphorylation of the inhibitory site Ser-9/21.

Figure 7b shows that phosphorylation of neuronal GSK3α at Ser-21 and GSK3β at the homologous Ser-9 was significantly reduced at 240 min of glutamate treatment. Thus, results of western blot and quantitative proteomic analyses indicated dephosphorylation of GSK3α/β as a cellular event induced by overstimulation of glutamate receptors (Fig. 7, Table 1 and S2). Since Akt inactivates GSK3α/β by direct phosphorylation of Ser-21/Ser-9^41^, our results suggest inactivation of Akt and consequential activation of GSK3α/β in excitotoxicity. Indeed, phosphorylation levels of Akt at Thr-308 and Ser-473, which govern Akt activation, were slightly reduced at 15 and significantly reduced at 240 min of glutamate treatment (Fig. 7b), indicating inhibition of Akt in excitotoxicity. In summary, the time-dependent changes in phosphorylation states of Map1b and Tau reflects the dynamics of perturbation of the Akt-GSK3α/β signalling pathway in excitotoxicity.

### Casein kinase 2 was activated in neurons undergoing excitotoxic cell death

The phosphosite sequences in Sept2, Ctnna2, Eif5b and Huwe1 conform with the optimal phosphorylation sequence of CK2 (Fig. 8a)^44,45^, suggesting that CK2 is a potential upstream kinase targeting these sites. Our prediction is further supported by previous studies demonstrating CK2 phosphorylation of Sept2 and Ctnna2 in cells and in vitro^46–48^. The dynamic changes of phosphorylation levels of the phosphosites of these four proteins predict that neuronal CK2 is activated as early as 5 min of glutamate treatment (Fig. 6). To validate this prediction, we immunoprecipitated CK2 from lysates of untreated neurons and those of neurons treated with glutamate at the designated time points and determined its specific kinase activity. Figure 8b shows that glutamate overstimulation led to activation of CK2 as early as 5 min and the activation was sustained till 240 min. Since activation of neuronal CK2 in excitotoxicity has not been documented, our findings illustrate how proteomic data can provide clues to the discovery of how a protein kinase is regulated in excitotoxicity.

## Discussion

The major take-home message of our findings is that excitotoxic neuronal death is the result of cumulative actions of a number of active neurotoxic pathways as well as inactivation of neurotrophic pathways. To fully decipher the signalling mechanism directing excitotoxic neuronal death, researchers need to investigate how these pathways interplay temporally to direct cell death. Our findings, as depicted in Fig. 6, provide researchers with a conceptual framework for future investigations to decipher the cell signalling mechanism of excitotoxicity. Since our results indicate significant changes in the activation states of neuronal Erk1/2, CK2, Akt and GSK3α/β in excitotoxicity, the discussion below focuses on their regulation and their potential roles in neuronal death.

### How are Erk1/2 and CK2 activated in excitotoxicity?

Activation of Erk1/2 contributes to neuronal death^28,39,49,50^ and ischaemic brain damage^28,51^. It is well established that Ras, Raf and MEK form the major upstream signalling pathway to activate Erk1/2 in cells. In neurons, overstimulation of synaptic NMDA receptor activates the Ras/Raf/MEK/Erk1/2 signalling cascade. This activation is mediated by the calmodulin-dependent synaptic RasGRF1, which facilitates the exchange of guanine nucleotide bound to Ras, leading to its activation^52^. The GAPase activator protein SynGAP1, however, inactivates Ras by promoting hydrolysis of the bound GTP. Since formation of stable complexes with Tau prevents SynGAP1 from locating to the synapse to suppress Ras activation^28^, future investigation should focus on examining if tau participates in activation of Erk1/2 by forming complexes with SynGAP1 in neurons in excitotoxicity.

CK2 is a tetramer formed by two α subunits with kinase domain, and two β subunits with protein substrate-docking motifs. However, unlike the “conventional” protein kinases, CK2 α subunit is constitutively active and little is known about the regulatory mechanism of its kinase activity^53^. Since we found that glutamate overstimulation induces activation of neuronal CK2, future investigation on how CK2 is activated in excitotoxicity will unveil its regulatory mechanism and its role in governing neuronal survival.

### How does glutamate overstimulation perturb the Akt/GSK3 signalling pathway in excitotoxicity?

Previous studies revealed multiple upstream regulatory mechanisms governing inactivation of Akt and activation of GSK3α/β in neurons^18,54^. Akt is activated by phosphorylation at Thr-308 in the activation loop and Ser-473 in the C-terminal region^55^. The phosphorylation events are governed by binding of Akt and the upstream regulatory kinases to the second messenger phosphatidylinositol-3,4,5-trisphosphate generated by phosphatidylinositide-3-kinase (PI3-kinase). We and other researchers discovered multiple cellular events that suppress neuronal Akt activation by these well-established upstream mechanisms in excitotoxicity. First, in excitotoxicity, the overactivated calpains cleaves the metabotropic glutamate receptor 1α (mGluR1α) to remove the C-terminal tail critical for binding and activation of PI3-kinase^56^. The cleavage abolishes the mGluR1α-mediated activation of PI3-kinase and in turn suppresses activation of Akt. More importantly, blockade of calpain cleavage of mGluR1α could protect against excitotoxic neuronal death, indicating that the calpain/mGluR1α/PI3K/Akt pathway is a key neurotoxic pathway in excitotoxicity. Second, the overactivated calpain also cleaves the protein tyrosine kinase c-Src at a site near its N-terminus in neurons undergoing excitotoxic cell death^18^. The cleavage generated a truncated c-Src fragment that functions as a pro-death protein kinase, which directs neuronal death in part by suppressing the activation of Akt. Furthermore, blockade of calpain cleavage of c-Src prevents inactivation of Akt and in turn protects against excitotoxic neuronal death, indicating the calpain/c-Src/Akt pathway as a key neurotoxic signalling pathway.

Besides activation by dephosphorylation of Ser-9 and Ser-21, GSK3α/β phosphorylation of their substrates can be facilitated by direct binding to substrate-docking proteins. GSK3β was found to directly bind to the p25 protein generated by calpain-mediated truncation of the activator protein p35 of the cyclin-dependent kinase 5 (Cdk5)^57^. The binding facilitates aberrant hyper-phosphorylation of Tau by GSK3β and contributes to the development of neurodegenerative phenotypes. Since we found that neuronal calpains are overactivated in excitotoxicity and calpain cleavage of neuronal p35 to p25 is a key event in excitotoxicity (Figure S3)^38^, the calpain/p25/GSK3β signalling pathway is a potential neurotoxic pathway contributing to activation of GSK3β and its aberrant phosphorylation of Tau in neurons undergoing excitotoxic cell death.

## Experimental procedures

### Excitotoxicity model and treatment of cultured primary cortical neurons under various experimental conditions

To induce excitotoxicity of the cultured primary cortical neurons, 100 μM glutamate was prepared as an aqueous solution in NB/B27 media and added to the cultures on DIV7^18,58^ for time intervals of 5, 15, 30, 60, 240 and 480 min.

### Cell viability assay

Cell viability was determined from primary cortical neurons (seeded in 24-well plates) using the MTT assay. MTT solution (5 mg/ml dissolved in RPMI/1640 medium phenol red free) equal to 10% (v/v) was added to each experimental well and after 30 min incubation, the culture medium was removed and 200 µl DMSO was added per well to dissolve the formazan crystals formed and a 100-µl aliquot from each well transferred to a separate well in a 96-well plate. The 570 nm absorbance was measured using FLUOstar Optima plate reader (BMG Lab Technologies, Durham, NC). Cell viability was expressed as a percentage of the control cells.

### Preparation of neuronal lysates

The glutamate treated cortical neurons (seeded in six-well plates) were lysed in ice-cold RIPA buffer (50 mM Tris pH 7.0, 1 mM EDTA, 5 mM EGTA, 1 mM dithiothreitol, 10% (v/v) glycerol, 1% Triton X-100, 0.01% SDS, 150 mM NaCl, 50 mM NaF, 40 mM sodium pyrophosphate, 0.5 mM Na_3_VO_4_, 50 mM β-glycerol phosphate, 0.2 mg/ml benzamidine, 0.1 mg/ml phenyl methyl sulfonyl fluoride (PMSF), EDTA-free protease and phosphatase inhibitors cocktail (Roche, Indianapolis, IN, USA)). The lysed cells were harvested, transferred to a centrifuge tube and centrifuged at 12,500 × *g* for 10 min at 4 °C. The supernatant was collected into a new tube and stored at −80 °C for further analysis. Total protein concentration determined by BCA protein assay (Pierce-Thermo Scientific).

### Acetone precipitation of neuronal lysates

Neuronal lysates were mixed with freezer-cold acetone (1:5 v/v) and incubated at −20 °C overnight to precipitate proteins. The tubes were centrifuged at 12,500 × *g* for 15 min at 4 °C. The protein precipitates were washed with freezer-cold acetone, centrifuged at 12,500 × *g* for 5 min. After removal of the supernatant, the acetone precipitated protein pellets were air-dried completely and resuspended in the appropriate buffer for subsequent analysis.

### Generation of tryptic digests of neuronal lysates for stable isotope dimethyl labelling

Acetone precipitated protein lysates were resuspended in 8 M urea in 50 mM triethyl ammonium bicarbonate (TEAB) buffer (pH 8.0) by alternate vortex and sonication, and protein concentration determined by BCA assay. 200 μg of resuspended proteins from control lysates (5× aliquots) and 200 µg of proteins from glutamate treated neuronal lysates (1× aliquot each for the lysates from neurons treated with glutamate for 5, 15, 30, 60 and 240 min) were reduced with 10 mM tris-(2-carboxyethyl)-phosphine for 45 min at 37 °C with constant agitation. The reduced protein samples were alkylated with 55 mM iodoacetamide in the dark for 30 min. Samples were diluted to 1 M urea using 25 mM TEAB and digested with sequencing grade-modified trypsin (1:50 w/w) with overnight agitation at 37 °C. The digested samples were acidified to 1% (v/v) formic acid. Each digested sample was subjected to the solid phase extraction (SPE) clean-up procedures, using a 60 mg Oasis HBL cartridge (Waters). Briefly, the cartridge was pre-washed with 80% acetonitrile (ACN) containing 0.1% trifluoro acetic acid (TFA) in Milli-Q water prior to equilibration with 0.1% TFA in Milli-Q water. After loading the trypsin-digested peptides to the pre-washed and equilibrated columns, the columns were washed with 0.1% TFA in water and the bound tryptic peptides were eluted with 800 µl of elution buffer containing 80% acetonitrile, 20% Milli-Q water and 0.1% TFA. The eluted peptides were partially reduced in volume by Speedy-Vac centrifugation and then freeze-dried.

### Stable isotope dimethyl labelling of tryptic peptides

Stable isotope dimethyl labelling was performed according to the protocol described earlier by Boersema et al.^59^. In brief, the freeze-dried tryptic peptides were re-dissolved in 100 mM TEAB. At this stage, micro BCA quantitation assay of the samples were carried out to ensure equal amounts (25 µg each) of dissolved digested peptides derived from the control and the glutamate-treated neuronal lysates were used for labelling. For every 25 µg of peptides, 4 μl of normal formaldehyde, CH_2_O [4% (v/v)] (light label) and 4 μl of deuterated formaldehyde, CD_2_O [4% (v/v)] (medium label) were used for reductive amination of the α-amino group of the free N-termini and the ɛ-amino group of lysine residues of the tryptic peptides derived from control and treated neuronal lysates, respectively. To each tube, 4 μl of 0.6 M sodium cyanoborohydride (NaBH_3_CN) was added to initiate the labelling reaction at room temperature for 60 min. To stop the labelling reaction, 16 μl of 1% (v/v) ammonia in Milli-Q H_2_O was added to each tube and the samples were mixed briefly. Formic acid (8 µl) was added to further quench the reaction. The reaction mixtures were left on ice. At this stage, the differentially labelled samples were mixed at a ratio of light: medium = 1:1 (w/w). An aliquot (10 µl) of the mixture was taken for analysis by liquid chromatography tandem-mass spectrometry (LC-MS/MS) to compare the abundance of the dimethyl labelled tryptic peptides derived from cellular proteins from the control and glutamate treated neurons. The remaining portion was subject to SPE clean-up, freeze-dried and used for TiO_2_ phosphopeptides enrichment.

### Enrichment of phosphopeptides by TiO_2_ microcolumn chromatography

Phosphopeptides in the freeze-dried samples were enriched by TiO_2_ by following the procedures described by Thingholm et al.^60^. In brief, the freeze-dried samples were reconstituted with 80 µl of the DHB (2,5-dihydroxybenzoic acid) loading buffer containing 0.2 g/ml DHB in the wash buffer (80% ACN, 3% TFA). TiO_2_ loaded micro-columns were washed with 30 µl of 100% ACN before sample loading and the columns were centrifuged at 500 × *g*. After sample loading, columns were washed sequentially with 20 µl of DHB loading buffer and 60 µl of wash buffer. Samples were eluted in clean microfuge tubes with 80 µl of Elution Buffer containing 0.5% (v/v) ammonia in Milli-Q H_2_O. Elution was continued with 2 µl of 30% ACN in Milli-Q water. Eluents were acidified with 1 µl of pure formic acid per 10 µl of eluent to obtain pH of 2–3. Samples were concentrated using a centrifugal evaporator to ~20–30 µl before analysis by LC-MS/MS. Results of the analysis revealed the dynamic changes of neuronal phosphoproteome associated with glutamate-induced excitotoxicity.

### LC-MS/MS analysis

The resultant tryptic peptides (mixed labelled peptides with and without TiO_2_ enrichment) were analysed on a LTQ Orbitrap Elite (Thermo Scientific) mass spectrometer coupled to an Ultimate 3000 nano LC system (Dionex) equipped with an Acclaim Pepmap nano-trap column (Dionex – C18, 100 Å, 75 µm × 2 cm) and an Acclaim Pepmap analytical column (Dionex C18, 2 µm, 100 Å, 75 µm × 15 cm). The peptide mixture was loaded onto the trap column isocratically with 3% (v/v) ACN in 0.1% (v/v) formic acid at a flow rate of 5 µl/min before the enrichment column was switched in-line with the analytical column. The bound peptides were eluted by a gradient made up of Solvent A (0.1% (v/v) formic acid) and Solvent B (0.1% (v/v) formic acid in ACN). The flow gradients were (i) 3–12% of Solvent B for 1 min, (ii) 12–35% of Solvent B for 20 min, (iii) 35–80% of Solvent B for 2 min and (iv) elution with 80% of Solvent B was sustained for 2 min. The column was equilibrated with 3% of Solvent B for 7 min prior to the next sample injection. The mass spectrometer was operated in the data-dependent mode with nano ESI spray voltage of +2.0 kV, capillary temperature of 250°C and S-lens RF value of 60%. The data dependent mode refers to the procedure whereby spectra were acquired first in positive mode with full scan MS spectra scanning from *m*/*z* 300–1650 in the FT mode at 240,000 resolution followed by rapid collision-induced dissociation (rCID) in the ion trap. The top 20 of the most intense peptide ions with charge states ≥2 were isolated and fragmented using normalized collision energy of 35 and activation Q of 0.25.

### Processing of raw mass spectrometric data

Raw mass spectrometric data were processed using Proteome Discoverer 2.1 (PD 2.1) (Thermo Scientific, USA) with Mascot (Matrix Science version 2.4) search algorithm against mouse Swissprot database. Search parameters were: (i) precursor mass tolerance = 10 ppm, (ii) fragment mass tolerance: 0.6 Da with signal-to-noise ratio of 1.0 and (iii) mass precision = 2 ppm. Carbamidomethyl cysteine was set as fixed modification and oxidation of methionine was set as variable modifications. Two-plexed dimethylation (C_2_H_6_, C_2_H_2_D_4_) was selected as the quantification method for dimethyl modification at the N-terminus of the peptide and lysine. Trypsin was used as the cleavage enzyme with a maximum of two missed cleavages allowed. Results were set to reflect a maximum of 1% FDR with ≥2 peptides and at least one unique peptide required for positive identifications. For phosphorylation site prediction, the ptmRS node in PD 2.1 was used, it contains the phospho-site localization algorithm^61^. The relative expression patterns of identified proteins or the phosphorylation status of phosphoproteins were determined based on the relative intensities of the reporter ions of the corresponding peptides using the quantitative node in PD 2.1. The mass spectrometry proteomics data have been deposited to the ProteomeXchange Consortium via the PRIDE^62^ partner repository with the dataset identifier PXD008353.

### Analysis of the proteomic data

Processed raw data were exported in MS Excel format from PD 2.1. Global proteomic and phosphoproteomic data were analysed separately. For the global proteomic data, neuronal proteins identified with peptide ratios (medium to light, M/L) with any missing quantification channel were avoided to minimise protein quantitation error. On the other hand, for phosphopeptide ratios with any missing quantification channel, values were replaced with a minimum intensity (maximum and minimum fold change = 100 and 0.01, respectively). The Perseus version 1.5.6.0 software was used for further data analysis^63^. For the global proteomic data, M/L ratios and the corresponding protein groups were imported in Perseus with all other columns derived from PD 2.1. For the phosphoproteome data, M/L ratios and the corresponding phosphopeptide groups with modifications were imported in Perseus with all other columns derived from PD 2.1. M/L ratios for biological replicates were grouped together, log2-transformed and normalized to column median. One-sample *t*-test was performed to identify neuronal proteins and phosphoproteins with significant changes in abundance and/or phosphorylation levels at all different time points. Because of the relatively low number of biological replicates (*n* = 3 for 5–60 min samples or *n* = 6 for 240 min samples), we chose a 2.5-fold changes (increase or decrease) in the mean normalised ratios as the criteria for significant perturbations. In our analysis, identified neuronal proteins and phosphopeptides with *p* ≤ 0.05 and mean normalized log2 ratio−SD > 1.32 or mean normalized log2 ratio + SD < −1.32 (i.e. 2.5-fold increase or decrease, respectively) were considered to have undergone significantly changes in abundance and/or phosphorylation levels in response to glutamate overstimulation. These proteins are referred to as the significantly changed neuronal proteins.

### Signalling pathway and network analysis using the Ingenuity Pathway Analysis software

Proteomic data were analysed using QIAGEN’s Ingenuity Pathway Analysis (IPA, QIAGEN Redwood City, www.qiagen.com/ingenuity) software for prediction of the signalling pathways and interaction networks^64^. The regulated neuronal proteins and phosphoproteins, accession numbers and median medium to light ratios (converted to fold changes by the software) were input into IPA using the core analysis platform. The core analysis matched proteins in the uploaded data set with those in the Ingenuity Knowledge base. The statistical significance of each network or list was determined by IPA using a Fisher exact test (*p* < 0.05). IPA was also used to construct network of interacting proteins. The current knowledge available on genes, proteins, normal and disease cellular processes, signalling and metabolic pathways were used from the IPA database for pathway construction.

### Prediction of the upstream protein kinases directly phosphorylating some of the phosphosites identified in the proteomic study

For the neuronal proteins exhibiting increases in phosphorylation in response to glutamate overstimulation, we adopted a standard bioinformatic procedure to predict their upstream protein kinases. This procedure involves (i) following the temporal changes in the abundance and phosphorylation states of the identified changed neuronal proteins and selecting only the phosphosites exhibiting time-dependent increase in phosphorylation level for further analysis, (ii) comparing the amino acid sequences of the selected identified phosphosites with the optimal phosphorylation sequences of known protein kinases^34^ and (iii) in-depth analysis of the relevant publications on the potential upstream protein kinases targeting these phosphosites in various cell types. Protein kinases predicted by this procedure as the upstream kinases directing phosphorylating the selected phosphosite are presented in Fig. 6. In Table 1, our predictions are compared with the predictions listed in NetworKIN and PhosphoSitePlus^65,66^.

### Measurement of the kinase activity of CK2 isolated from untreated neurons and neurons treated with glutamate

The relative amounts of CK2 in glutamate-treated and untreated neurons were first determined by Western blotting. Aliquots of neuronal lysates with relatively equal amounts of CK2 were then used for immunoprecipitation to isolate CK2 with an anti-CK2 antibody conjugated with Protein A-agarose and CK2 kinase activity assay was performed using a peptide substrate as described earlier^67^. Briefly, anti-CK2α antibody (cat. #2656, Cell Signalling Technology) was added to washed protein A-agarose (1:50; v/v) and incubated for 2 h in a rotator in the cold room (4 °C) and washed 2× with cold PBS and 1× with lysis buffer to remove unconjugated excess antibodies. Anti-CK2α-conjugated protein A-agarose beads were then added to neuronal lysates (1:10; v:v) and incubated for 2 h in the cold room in a rotator. Following incubation agarose beads were washed 2× lysis buffer and 2× with wash buffer (50 mM HEPES pH 7.4, 150 mM NaCl, 10% glycerol, 1 mM DTT and 0.1% Tween-20) prior to the kinase reaction. CK2 kinase activity was assayed using radiolabelled ^32^P-ATP and synthetic peptide substrate CK2-tide (RRRDDDSDDD-NH_2_). Activity assay was conducted in the presence of 200 µM CKtide peptide, 5 mM MgCl_2_, 200 µM [γ-^32^P] ATP for 10 min at 30 °C. Phosphotransferase activity was quenched by spotting 15 μl onto P81 phosphocellulose paper (Whatman, GE Healthcare), followed by repeated washes in 1% (v/v) phosphoric acid. ^32^P transfer was quantified by liquid scintillation counting (Perkin Elmer). Relative CK2 activity in different neuronal lysates (glutamate treated for 5–240 min) was expressed as percentage of control (untreated neuronal lysates).

## Supplementary information

Supplemental Information Part 1

Suppemental Table S1

Supplemental Table S2

Supplemental Table S3
