# Supplementary material for: Quantitative proteomic analyses of dynamic signalling events in cortical neurons undergoing excitotoxic cell death
Source: Cell Death Dis. 2019 Mar 1;10(3):213. doi: 10.1038/s41419-019-1445-0 (PMC6397184; doi:10.1038/s41419-019-1445-0)
Supplement: Supplementary file 1 — Supplemental Information Part 1 [file 41419_2019_1445_MOESM1_ESM.pdf]

Quantitative Proteomic Analyses of Dynamic Signalling Events in Cortical Neurons Undergoing Excitotoxic Cell Death

Hoque, *et al.*

SUPPLEMENTARY INFORMATION PART 1

- **Supplemental Experimental Procedures**
- **Supplemental Results**
- **Supplemental Figures**

  Figure S1. Morphological features of cultured primary cortical neurons at seven days in vitro (DIV7)

  Figure S2. Time-dependent morphological changes of cultured primary cortical neurons in response to glutamate treatment

  Figure S3. Necrosis involving the activation of calpains is the major cell-death signalling mechanism in neurons treated with glutamate for 240 min

  Figure S4. Heatmap depicting time-dependent changes in abundance of selected neuronal proteins

  Figure S5. Multi-scatter plot showing the correlation between replicates of global proteomic changes at different time points

  Figure S6. Multi-scatter plot showing the correlation between replicates of phosphoproteomic changes at different time points

  Figure S7. Volcano plots showing the temporal changes in abundance of identified phosphopeptides in neurons at different time points after glutamate treatment

  Figure S8. Top canonical pathways and disease and function annotations identified by IPA

  Figure S9. Top-ranked disease and function interaction networks identified by IPA

  Figure S10. Western blot analysis of the CK2 expression in neurons and detection of CK2 immunoprecipitated from neuronal lysate

- **Supplemental References**

Supplemental Experimental Procedures

Materials

Neurobasal medium, B-27 supplement, GlutaMAX-I, Penicillin, Streptomycin and RPMI 1640 medium were purchased from GIBCO (Rockville, MD, USA). Trypsin, soybean trypsin inhibitor, sodium pyruvate, L-glutamine, glutamate, DMSO, DNase, poly-D-lysine, iodoacetamide, formic acid, 2,5-dihydrobenzoic acid, sodium cyanoborohydride, formaldehyde solution were purchased from Sigma (St Louis, MO). Bond-Breaker TCEP solution from ThermoFisher Scientific, Titansphere (10µm) from GL Sciences, Oasis HLB 1cc cartridges from Waters, sequence grade trypsin from Promega and formaldehyde-D2 were sourced from Cambridge Isotope Laboratories. Anti-Akt, phospho-Akt (S473), phospho-Akt (T308), anti-p44/42 MAPK (Erk1/2), phospho-p44/42 MAPK (Erk1/2), anti-GSK3αβ and phospho-GSK3αβ (Ser21/9), anti-CK2α, antibodies were sourced from Cell Signalling and used at 1:1000 dilution unless otherwise mentioned.

Culture of primary mouse cortical neurons

All experiments involving animals were approved by the University of Melbourne Animal Ethics Committee (Licence number: 161394.1) and were performed in accordance with the Prevention of Cruelty to Animals Act 1986 under the guidelines of the National Health and Medical Research Council Code of Practice for the Care and Use of Animals for Experimental Purposes in Australia. For preparation of primary cortical neuronal cultures, embryos were collected from pregnant mice (gestational day 15-16) after they were euthanized by CO_2 asphyxiation. The cortical region was aseptically micro-dissected out of the brains of the embryos, free of meninges and dissociated in Hanks Balanced Salt Solution (HBSS) (9.5 g/l Hanks Balanced Salt, 7.4 mM glucose, 1 mM sodium pyruvate, 10 mM HEPES, 0.35 g/L sodium bicarbonate, 1.2 mM $MgSO_4$ and 3 mg/ml BSA). The suspended tissues were subjected to trypsin digestion (0.2 mg/ml trypsin and 0.04 mg/ml DNase in HBSS) at 37°C for 5 min and trypsin was inactivated by the addition of trypsin inhibitor (0.08 mg/ml). The suspension was centrifuged at 1000 × g for 5 min at room temperature. The tissue pellet was then subjected to mechanical trituration in HBSS solution containing 0.04 mg/ml DNase and 0.5 mg/ml trypsin inhibitor and then allowed to stand for 30 sec. The single-cell suspension was then transferred to a new sterile 50 ml tube and centrifuged for 5 min at 1000 × g at room temperature. The cell pellet was then re-suspended in warm (37°C) neurobasal medium supplemented with 2.5% B-27 supplement, 0.5 mM GlutaMAX-I, 100 IU/ml penicillin and 100 µg/ml streptomycin (NB/B27). Cells were counted and then plated at a density of 6×10^5 cells per well in 24-well plates and at 1.5×10^6 cells per well in 6-well plates pre-treated with 0.1 mg/ml Poly-D-lysine. All cultures were maintained at 37°C in a humidified incubator supplemented with 5% CO_2. After ~18-20 h, the medium was replaced with fresh NB/B27 culture medium. The cultured neurons were incubated for seven days (DIV7) with 50% of the medium replaced with fresh medium at DIV5. The DIV7 cultures were highly neuronal enriched and were used for the experiments.

Western blotting

Western blotting was performed as described previously [1]. Briefly, proteins (~30 µg in each well) in neuronal cell lysates were separated by 10 % SDS-PAGE gels using running buffer [25 mM Tris-HCl, 192 mM glycine, 10 % (w/v) SDS] for approximately 1.2 h at 150 V, and then transferred onto PVDF membranes. The membranes were then blocked with 3% (w/v) non-fat dry milk in Tris buffered saline with Tween-20 (TBST) (0.2 M Tris–HCl, pH 7.4, 1.5 M NaCl, and 0.1 % Tween-20). After blocking and washing with TBST, the membranes were probed with the primary antibodies overnight. The membranes were washed with TBST 3× before probing with horseradish peroxidase-conjugated goat anti-rabbit or goat anti-mouse IgG secondary antibodies (1: 5,000) (Monash Antibody Technologies Facilities, Victoria,

Australia) for 1 h at room temperature. Protein bands were visualized using chemiluminescence (ECL, Amersham Biosciences) according to manufacturer's instruction. Images were taken using Fuji film LAS-3000. For Western blot with anti-Tau, phospho-Tau and anti-CK2 antibodies, neuronal lysates were separated by SDS-PAGE on 12% gels and transferred to Immobilon-FL PVDF membranes (EMD Millipore). Membranes were blocked in PBS + 0.1% Tween-20 (PBST) with 2% (w/v) non-fat dry milk in PBST for 30 min at room temperature, washed and then incubated overnight with primary antibodies (1: 1000 dilutions in PBST). After washes with PBST, membranes were incubated with anti-rabbit or anti-mouse IgG secondary antibodies, fluorescently-labelled with IR680 or IR800 dye, for 1 h. Immunoreactive bands were visualised on an Odyssey® Infrared Imaging System with densitometry analyses determined using Image Studio Lite software (LI-COR Biosciences).

Histology methods

Primary cortical neurons were seeded at a density of 80,000 cells per well into a 12 well dish and grown on a poly-D-lysine treated 15 mm diameter glass coverslips. After 7 days *in vitro* culture, the cells were treated with 0 (untreated) and 100 µM glutamate for 240 min. At completion of treatment, cells were fixed in 4% paraformaldehyde (in PBS buffer) followed by cell permeabilization treatment (0.075% triton X in blocking buffer) and blocking (10% goat serum in PBS buffer) at room temperature. Primary antibodies were diluted in blocking buffer and incubated on cells overnight at 4°C then washed in PBS buffer followed by incubation in secondary antibodies for 60 min at RT, washed in PBS buffer then mounted on a glass slide with mounting media (prolong gold, Invitrogen). Slides were allowed to dry for at least 48 hr before imaging. Images were taken on a Zeiss axioscope epifluoresence microscope through either a 40×/0.75 or 63×/0.95 EC Plan-neofluor air objectives using a combination of excitation/emission filter sets for green (495nm/537nm), red (546nm/590nm) and DAPI (350/460nm), illuminated by a LED X-cite power source. Images were taken using a coolsnap ES^2 Monochrome CCD camera from Photometrics and ZEN2 pro software from Zeiss. Fluorescence images (8 to 10 slices) were taken using the z-stack module then processed using the modules for deconvolution and image projection into a single image using the ZEN pro software v 2.3 and exported as a .tiff file. Bright field images were taken by illuminating the cells using a Halogen 100W lamp. Antibodies used were: anti-MAP2 (mAb 1:500, Merck, Australia), anti-mAb-488 (1:500, Invitrogen, Australia), DAPI (Sigma, Australia), anti-GFAP (mAb 1:500, Cell Signalling Technology, Australia), anti-Tau (Rb, 1:2000, Dako Australia), anti-Iba1 (Rb, 1:500, Novachem, Australia). The purity of the DIV 7 neuronal cultures were assessed by staining cultures for neurons, microglia and astrocyte using specific marker antibodies. Over 90% of cells in the neuronal cultures were found to be neurons (data not shown).

Monitoring the cleavage of specific neuronal proteins by calpains and caspase 3 activated by treatments with glutamate, staurosporine (STS) and hydrogen peroxide (H_2O_2)

Lysates of primary cortical neurons (DIV7) with and without treatment with glutamate (100 µM), staurosporine (1 µM) or H_2O_2 (50 µM) for 240 min were analyzed by Western blotting. Lysates were run on 12% and 8% SDS_PAGE gels for caspase 3 and α-fodrin blots, respectively. The primary antibodies used were α-fodrin (1:1000, Cell Signalling #2122), caspase 3 (1:1000, Cell Signalling #9662) and α-tubulin (1:1000).

Supplemental Results

Neurons treated with glutamate for 240 min exhibited biochemical features of regulated necrosis but not apoptosis

Apoptosis and regulated necrosis are two major cell death mechanisms directing neuronal death (reviewed in [2]). One of the key events in apoptosis is activation of procaspase 3 by its upstream pro-apoptotic proteases such as caspase 9, which catalyze proteolytic processing of procaspase 3 (~32 kDa) to form the active caspase 3 consisting of the p17 and p12 subunits (reviewed in [3]). Thus, formation of the active caspase 3 is a biomarker of apoptosis. Regulated necrosis of neurons, however, is governed by over-activation of the calcium-dependent proteases calpains, which directly cleave many neuronal proteins to cause cell death.[2] Among them, α-fodrin and procaspase 3 are direct substrates of calpains.[4-6] Upon cleavage by calpains, α-fodrin forms two major proteolytic fragments of 145 kDa and 150 kDa while procaspase 3 forms a truncated fragment of 30 kDa.[5,7] Furthermore, calpain cleavage of procaspase 3 and procaspase 9 was found to prevent their activation by proteolytic processing to form the active caspase 3 and caspase 9.[4] Hence, both 145/150 kDa α-fodrin proteolytic fragments and 30 kDa truncated procaspase 3 fragment are biomarkers of necrosis.

To define the type of neuronal death triggered by glutamate over-stimulation, we monitored the activation states of procaspase 3 and α-fodrin in neurons with and without glutamate over-stimulation for 240 min. We included neurons treated with cytotoxic levels of staurosporine (STS) and hydrogen peroxide (H_2O_2) as controls because treatments of cultured primary neurons with both compounds were previously found to induce significant activation of calpains and slight activation of procaspase 3 [4,8]. Supplemental Figure S3 shows that treatment of cultured cortical neurons with glutamate, STS or H_2O_2 induced limited proteolysis of intact procaspase 3 to form the 30 kDa truncated procaspase 3 fragment. Presumably, the fragment was generated by calpain cleavage of the Ser-7/Val-8 peptide bond of intact procaspase 3 [4,5] defined previously by Wolf *et al.* [5] (Figure S3). In contrast, the active caspase 3 p17 subunit was not detectable in the lysate of neurons over-stimulated by glutamate while only a weak caspase 3 p17 subunit signal was detected in lysate of neurons treated with STS and H_2O_2. These procaspase 3 cleavage patterns suggest that all three treatments over-activated calpains in neurons and had little or no effect on procaspase 3 activation. This notion is further supported by the cleavage pattern of α-fodrin – all three treatments caused a significant reduction in intact α-fodrin accompanied by a significant increase in the abundance of the 145kDa/150kDa fragment generated by direct calpain cleavage of the Val-1175/Tyr-1176 and Gly-1230/Ser-1231 peptide bonds of intact α-fodrin [7]. Thus, results of Figure S3 indicate that necrosis associated with over-activation of calpains was the major type of cell death of neurons caused by glutamate over-stimulation and treatments with STS and H_2O_2 for 140 min. The results also reveal that glutamate over-stimulation did not cause apoptosis while treatment with STS and H_2O_2 only induced slight stimulation of the apoptotic signalling pathway.

Supplemental Figures

Figure S1

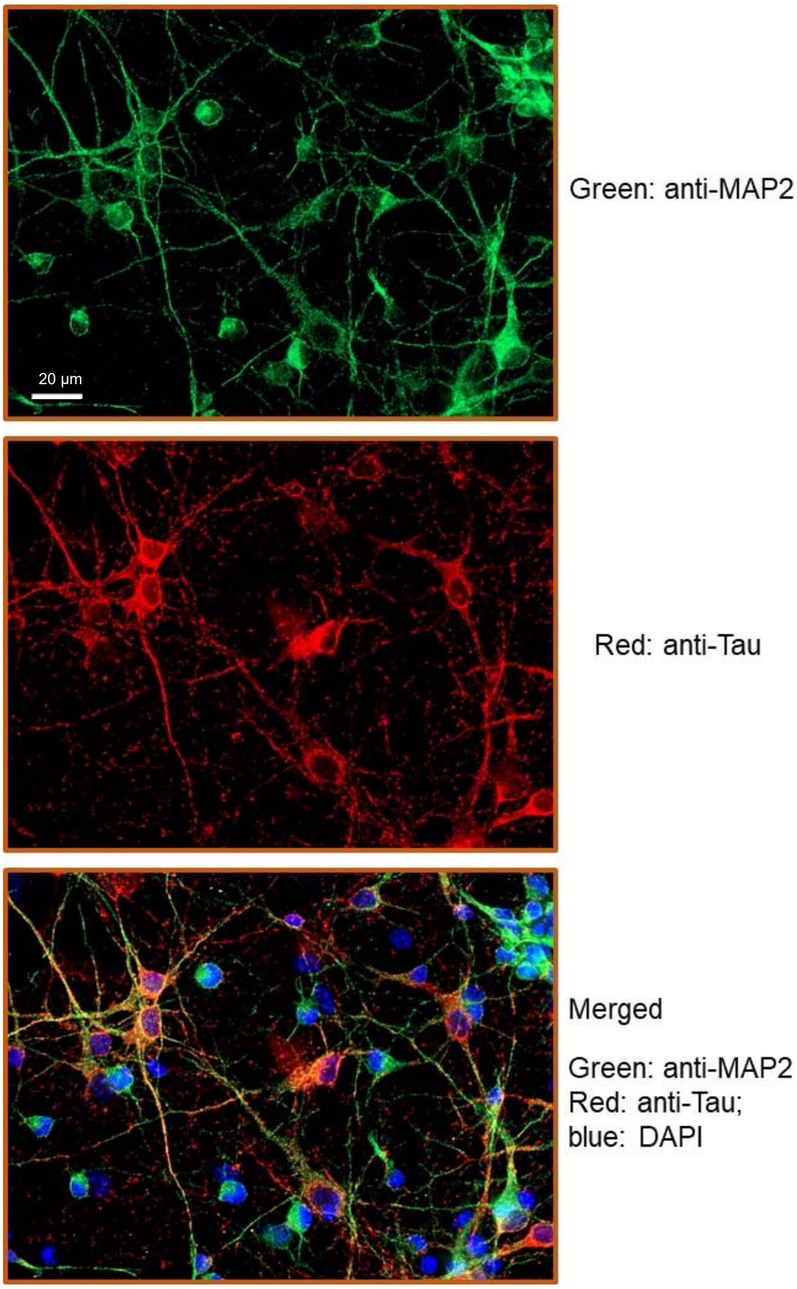

Figure S1. Morphological features of cultured primary cortical neurons at seven days in vitro (DIV7)

Untreated mouse primary cortical neurons were grown in culture for 7 days *in vitro* then fixed and immunostained for neuronal marker proteins, MAP2 (green) and Tau (red) proteins and the cell nucleus was stained using DAPI (blue) dye. Scale bar 20 µm.

Figure S2

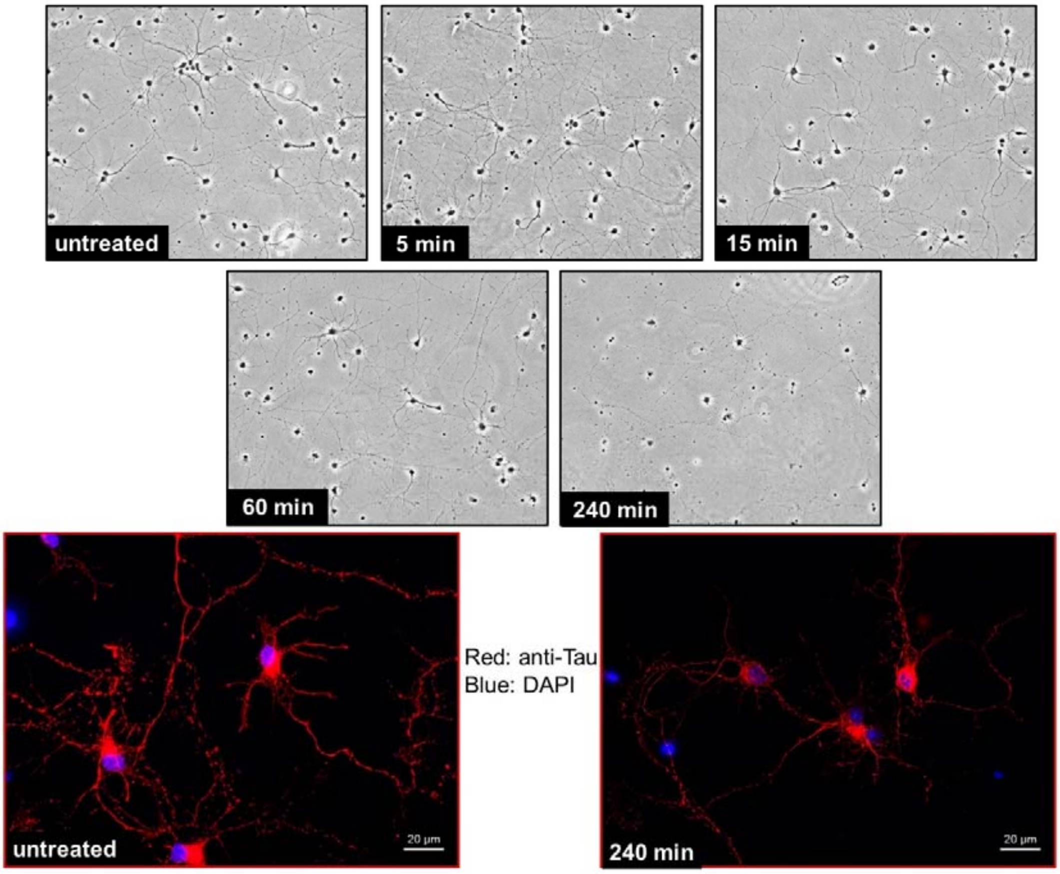

Figure S2. Time-dependent morphological changes of cultured primary cortical neurons in response to glutamate treatment

Glutamate treatment of cortical neurons causes morphological changes over time. Mouse primary cortical neurons were grown in culture for 7 days *in vitro* then treated with 100 µM glutamate for 5, 15, 60, and 240 min. Following treatment, cells were fixed and prepared for histological imaging (refer to the "Histology Methods" section). Phase contrast images of treated cultures (top panel of images) were taken to demonstrate how the presence of the neurite architecture morphology and cell soma shape in the untreated cells gradually diminished and dramatically altered especially in the 240 min treated cultures. Fixed cells were immunostained for the neuronal protein, Tau (red) and nucleus stained with DAPI (blue). The anti-Tau immunofluorescence intensity and extent of neurite architecture are dramatically diminished in the 240 min glutamate treated cultures compared to the untreated cultures (lower panels). Scale bar 20 µm.

Figure S3

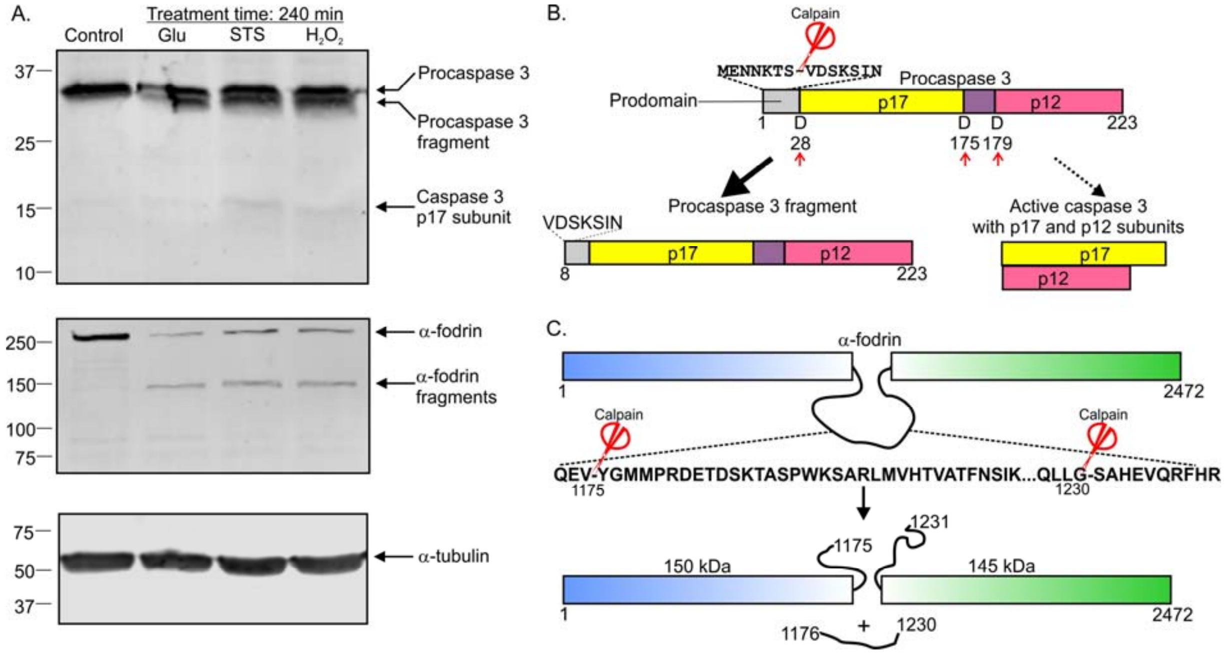

Figure S3. Necrosis involving the activation of calpains is the major cell-death signalling mechanism in neurons treated with glutamate for 240 min

A. Western blot analysis of crude lysates of control neurons and neurons treated with glutamate (100 μM), staurosporine (1 μM) and hydrogen peroxide (50 μM) for 240 min (labelled as Glu, STS and H_2O_2, respectively). The blots were probed with the antibodies against α-fodrin, caspase 3 and α-tubulin. **B.** Schematic depicting the products generated by calpain-mediated cleavage of procaspase 3 (~32 kDa) at the Ser-7/Val-8 peptide bond to generate the procaspase 3 fragment of ~30 kDa. In cells undergoing apoptosis, procaspase 3 is activated to form the activate caspase 3 fragments of 12-17 kDa. Results shown in panel A suggest that most of procaspase 3 molecules in neurons were cleaved by calpains to form the procaspase 3 fragment of ~30 kDa (indicated by the large solid black arrow). Only a very small portion of procaspase 3 molecules underwent proteolytic processing (indicated by the small dotted black arrow) at Asp-28, Asp-175 and Asp-179 (upward red arrows) by the upstream pro-apoptotic proteases to form the active mature caspase 3 consisting of the p17 and p12 subunit of ~12-17 kDa. As the epitope of the anti-caspase 3 antibody is mapped to the p17 subunit segment of intact procaspase 3, the antibody recognizes procaspase 3, procaspase 3 fragment, the p17 subunit but not the p12 subunit. **C.** Schematic depicting the products generated by calpain-mediated cleavage of α-fodrin at Val-1175/Tyr-1176 and Gly-1230/Ser-1231 peptide bonds. The numbers refer to the amino acid residue numbers of mouse α-fodrin (uniprot identifier P16546-1). Cleavage at the two sites is expected to convert intact α-fodrin (~285 kDa) to two fragments of 145-150 kDa.

Figure S4A

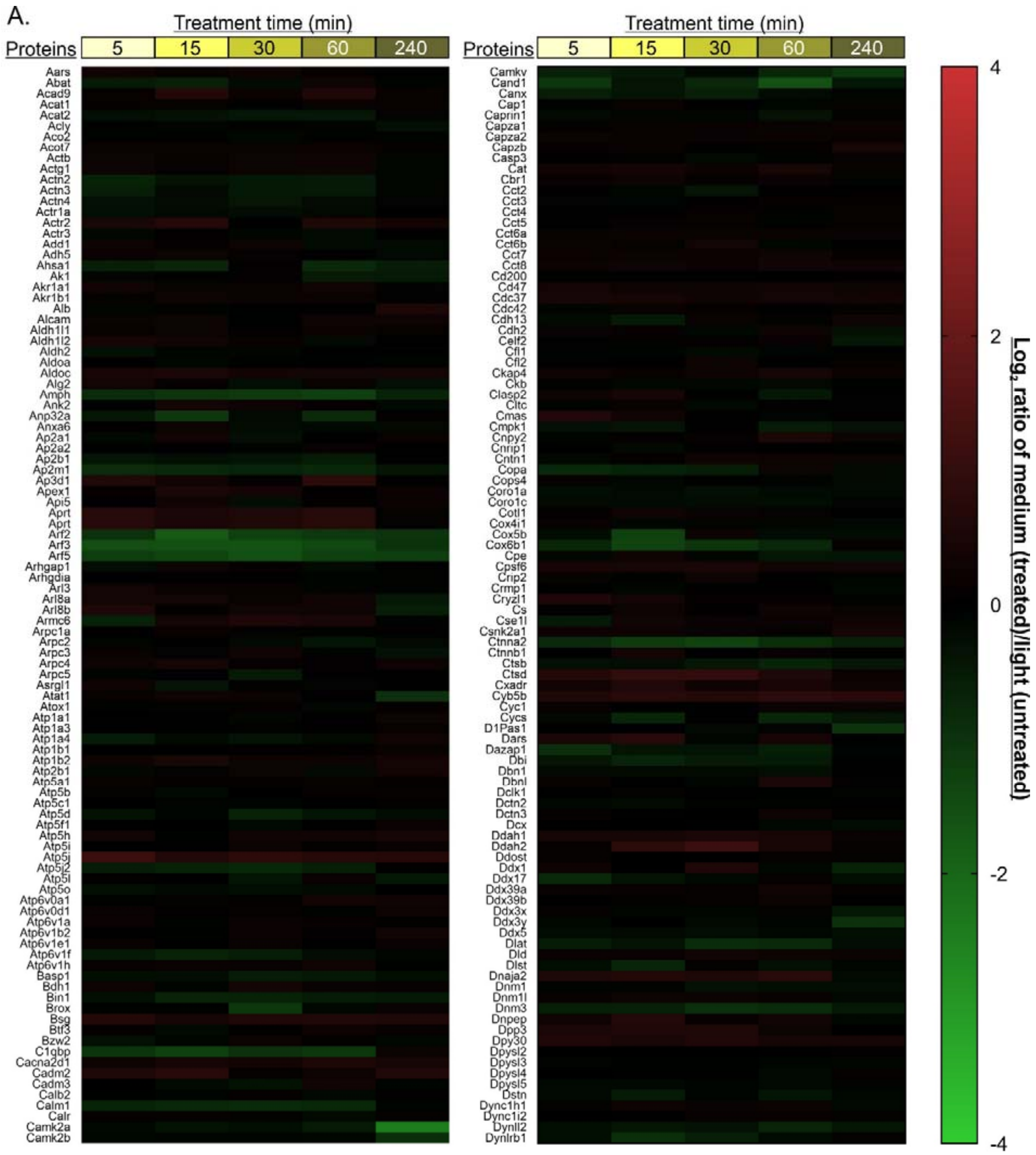
A.
Treatment time (min)
Proteins
5
15
30
60
240
Aars
Abat
Acad9
Acat1
Acat2
Acly
Aco2
Acot7
Actb
Actg1
Actn2
Actn3
Actn4
Actr1a
Actr2
Actr3
Add1
Adh5
Ahsa1
Ak1
Akr1a1
Akr1b1
Alb
Alcam
Aldh1l1
Aldh1l2
Aldh2
Aldoa
Aldoc
Alg2
Amph
Ank2
Anp32a
Anxa6
Ap2a1
Ap2a2
Ap2b1
Ap2m1
Ap3d1
Apex1
Api5
Aprt
Aprt
Arf2
Arf3
Arf5
Arhgap1
Arhgdia
Arl3
Arl8a
Arl8b
Armc6
Arpc1a
Arpc2
Arpc3
Arpc4
Arpc5
Asrgl1
Atat1
Atox1
Atp1a1
Atp1a3
Atp1a4
Atp1b1
Atp1b2
Atp2b1
Atp5a1
Atp5b
Atp5c1
Atp5d
Atp5f1
Atp5h
Atp5i
Atp5j
Atp5j2
Atp5l
Atp5o
Atp6v0a1
Atp6v0d1
Atp6v1a
Atp6v1b2
Atp6v1e1
Atp6v1f
Atp6v1h
Basp1
Bdh1
Bin1
Brox
Bsg
Btf3
Bzw2
C1qbp
Cacna2d1
Cadm2
Cadm3
Calb2
Calm1
Calr
Camk2a
Camk2b
Treatment time (min)
Proteins
5
15
30
60
240
Camkv
Cand1
Canx
Cap1
Caprin1
Capza1
Capza2
Capzb
Casp3
Cat
Cbr1
Cct2
Cct3
Cct4
Cct5
Cct6a
Cct6b
Cct7
Cct8
Cd200
Cd47
Cdc37
Cdc42
Cdh13
Cdh2
Celf2
Cfl1
Cfl2
Ckap4
Ckb
Clasp2
Cltc
Cmas
Cmpk1
Cnpy2
Cnrip1
Cntn1
Copa
Cops4
Coro1a
Coro1c
Cotl1
Cox4i1
Cox5b
Cox6b1
Cpe
Cpsf6
Crip2
Crmp1
Cryzl1
Cs
Cse1l
Csnk2a1
Ctnna2
Ctnnb1
Ctsb
Ctsd
Cxadr
Cyb5b
Cyc1
Cycs
D1Pas1
Dars
Dazap1
Dbi
Dbn1
Dbnl
Dclk1
Dctn2
Dctn3
Dcx
Ddah1
Ddah2
Ddost
Ddx1
Ddx17
Ddx39a
Ddx39b
Ddx3x
Ddx3y
Ddx5
Dlat
Dld
Dlst
Dnaja2
Dnm1
Dnm1l
Dnm3
Dnpep
Dpp3
Dpy30
Dpysl2
Dpysl3
Dpysl4
Dpysl5
Dstn
Dync1h1
Dync1i2
Dynll2
Dynlrb1
4
2
0
-2
-4
Log2 ratio of medium (treated)/light (untreated)

Figure S4B

B.

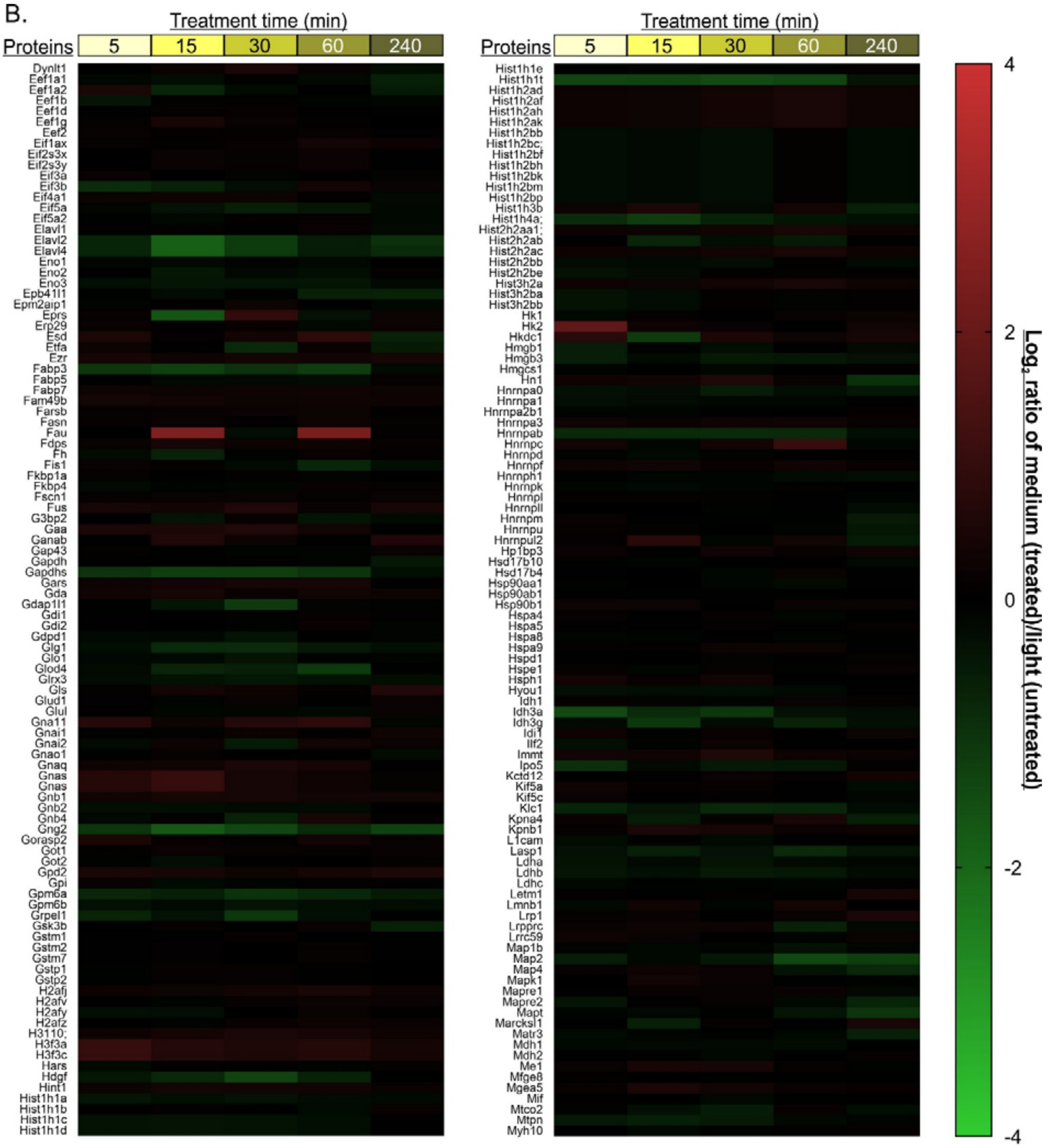

Treatment time (min)
Proteins
5
15
30
60
240
Dynlt1
Eef1a1
Eef1a2
Eef1b
Eef1d
Eef1g
Eef2
Eif1ax
Eif2s3x
Eif2s3y
Eif3a
Eif3b
Eif4a1
Eif5a
Eif5a2
Elavl1
Elavl2
Elavl4
Eno1
Eno2
Eno3
Epb41l1
Epm2aip1
Eprs
Erp29
Esd
Etfa
Ezr
Fabp3
Fabp5
Fabp7
Fam49b
Farsb
Fasn
Fau
Fdps
Fh
Fis1
Fkbp1a
Fkbp4
Fscn1
Fus
G3bp2
Gaa
Ganab
Gap43
Gapdh
Gapdhs
Gars
Gda
Gdap1l1
Gdi1
Gdi2
Gdpd1
Glg1
Glo1
Glod4
Glrx3
Gls
Glud1
Glul
Gna11
Gnai1
Gnai2
Gnao1
Gnaq
Gnas
Gnas
Gnb1
Gnb2
Gnb4
Gng2
Gorasp2
Got1
Got2
Gpd2
Gpi
Gpm6a
Gpm6b
Grpel1
Gsk3b
Gstm1
Gstm2
Gstm7
Gstp1
Gstp2
H2afj
H2afv
H2afy
H2afz
H3110;
H3f3a
H3f3c
Hars
Hdgf
Hint1
Hist1h1a
Hist1h1b
Hist1h1c
Hist1h1d
Treatment time (min)
Proteins
5
15
30
60
240
Hist1h1e
Hist1h1t
Hist1h2ad
Hist1h2af
Hist1h2ah
Hist1h2ak
Hist1h2bb
Hist1h2bc;
Hist1h2bf
Hist1h2bh
Hist1h2bk
Hist1h2bm
Hist1h2bp
Hist1h3b
Hist1h4a;
Hist2h2aa1;
Hist2h2ab
Hist2h2ac
Hist2h2bb
Hist2h2be
Hist3h2a
Hist3h2ba
Hist3h2bb
Hk1
Hk2
Hkdc1
Hmgb1
Hmgb3
Hmgcs1
Hn1
Hnrnpa0
Hnrnpa1
Hnrnpa2b1
Hnrnpa3
Hnrnpab
Hnrnpc
Hnrnpd
Hnrnpf
Hnrnph1
Hnrnpk
Hnrnpl
Hnrnpll
Hnrnpm
Hnrnpu
Hnrnpul2
Hp1bp3
Hsd17b10
Hsd17b4
Hsp90aa1
Hsp90ab1
Hsp90b1
Hspa4
Hspa5
Hspa8
Hspa9
Hspd1
Hspe1
Hsph1
Hyou1
Idh1
Idh3a
Idh3g
Idi1
Ilf2
Immt
Ipo5
Kctd12
Kif5a
Kif5c
Klc1
Kpna4
Kpnb1
L1cam
Lasp1
Ldha
Ldhb
Ldhc
Letm1
Lmnb1
Lrp1
Lrpprc
Lrrc59
Map1b
Map2
Map4
Mapk1
Mapre1
Mapre2
Mapt
Marcksl1
Matr3
Mdh1
Mdh2
Me1
Mfge8
Mgea5
Mif
Mtco2
Mtpn
Myh10
4
2
0
-2
-4
Log2 ratio of medium (treated)/light (untreated)

Figure S4C

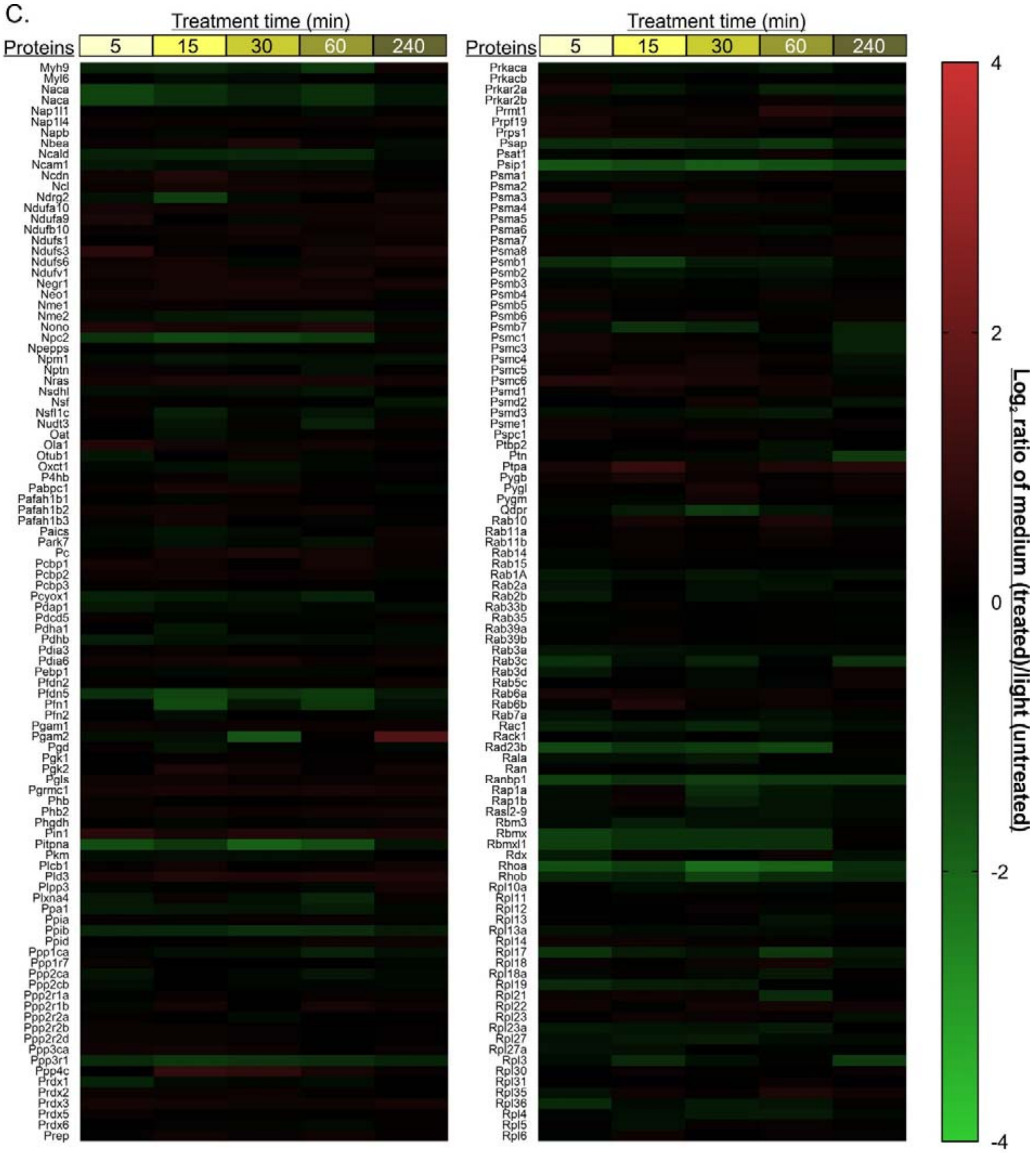
C.
Treatment time (min)
Proteins
5
15
30
60
240
Log2 ratio of medium (treated)/light (untreated)
4
2
0
-2
-4

Figure S4D

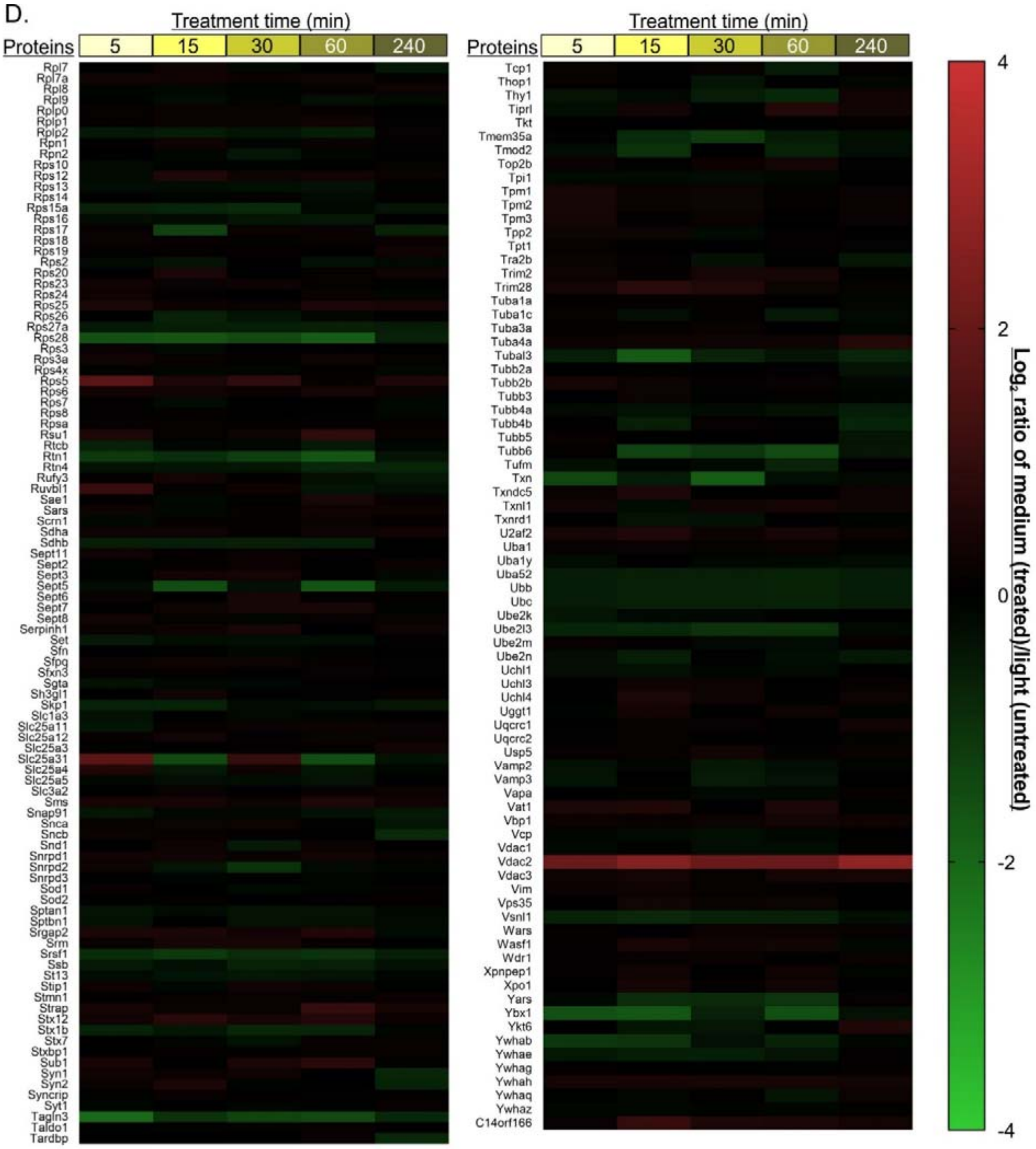

Figure S4. Heat maps depicting time-dependent changes in abundance of selected neuronal proteins

Temporal changes (5 min to 240 min) in abundance of selected neuronal proteins from global proteome data are presented in heat maps. Only neuronal proteins identified in at least two biological replicates in all the time points are selected for presentation. For the complete list identified proteins and corresponding abundance ratios, readers are requested to check Table S1.

Figure S5

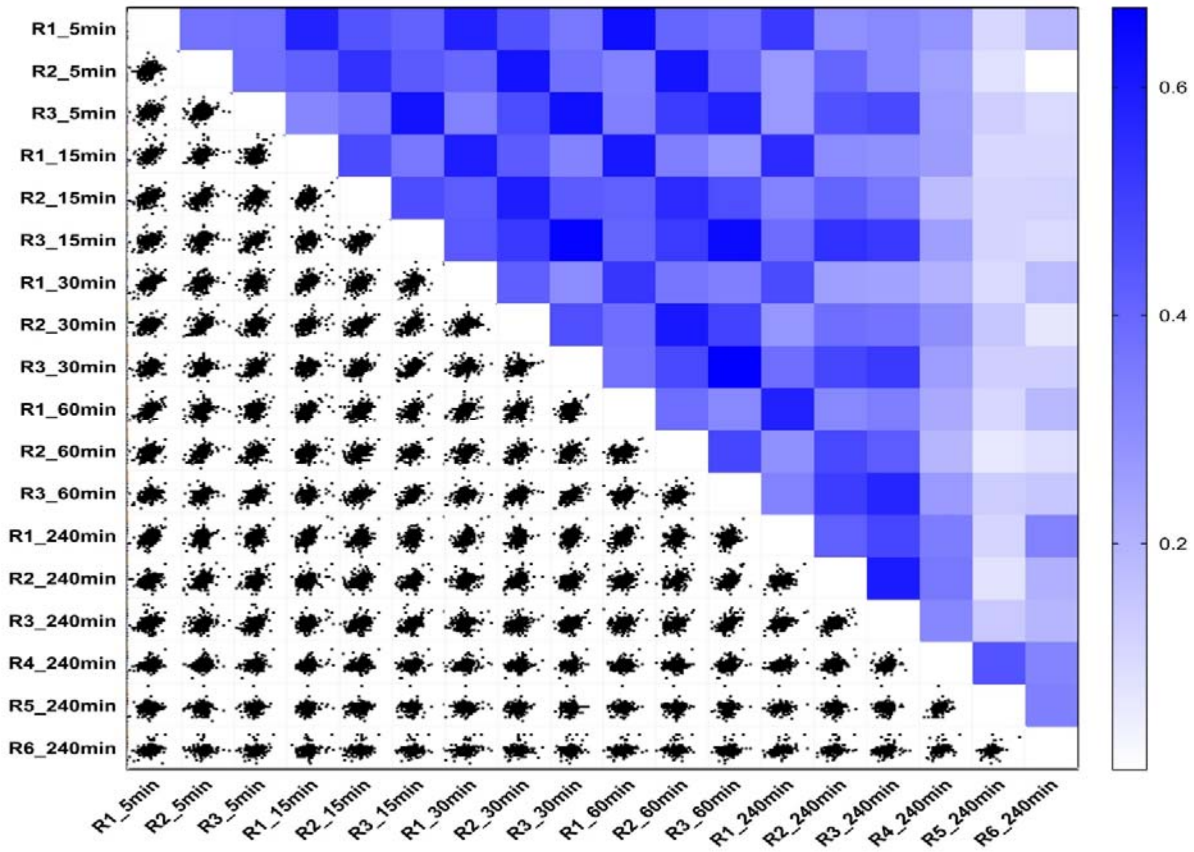

Figure S5. Multi-scatter plot showing the correlation between replicates of global proteomic changes at different time points

This plot depicts the variability in identified neuronal proteins over time. Darker blue represents higher Pearson's correlation between samples.

Figure S6

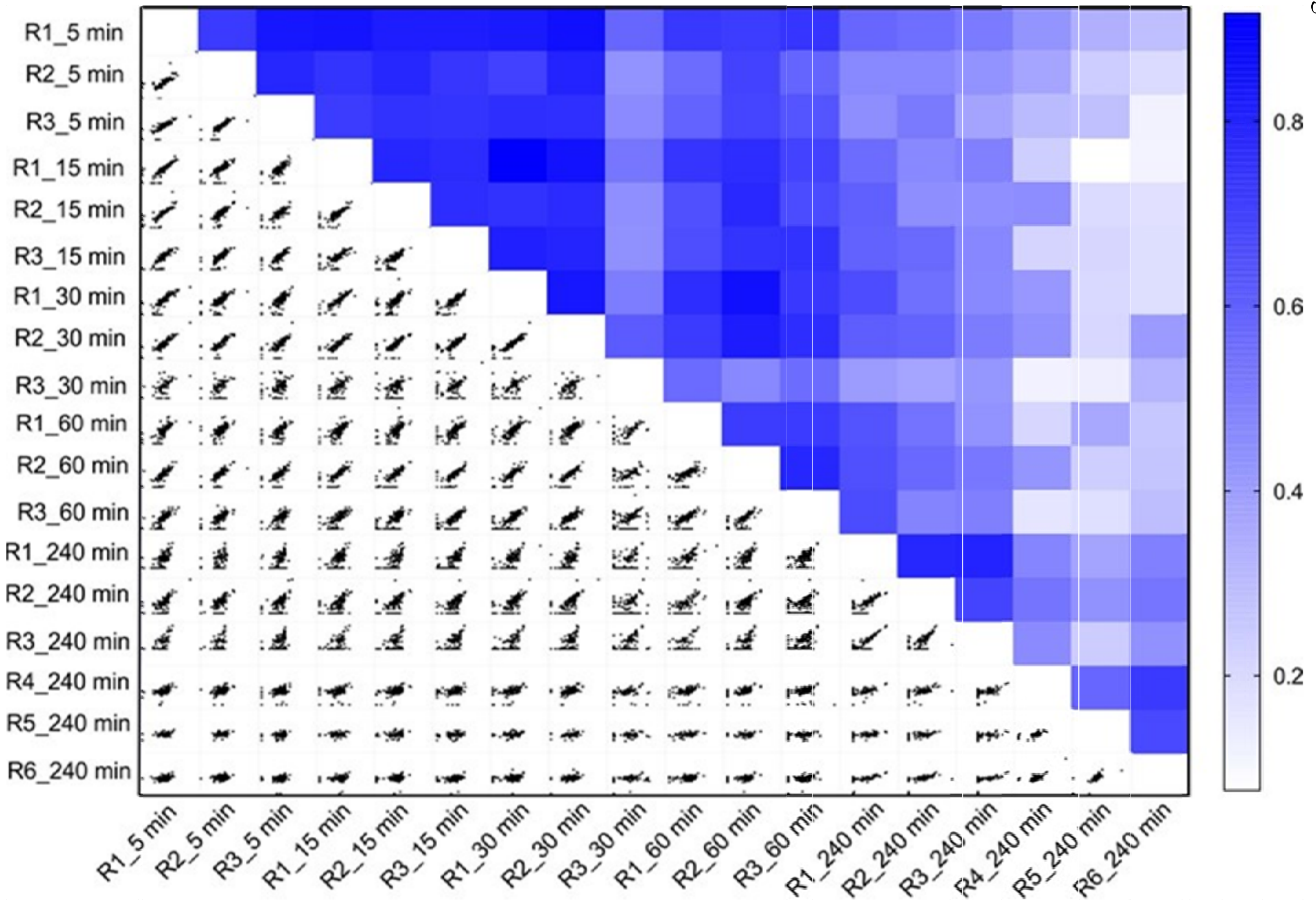

Figure S6. Multi-scatter plots showing the correlation between replicates of phosphoproteomic changes at different time points.
This scatter plot depicts the variability in identified neuronal phosphopeptides over time. Darker blue represents higher Pearson's correlation between samples.

Figure S7

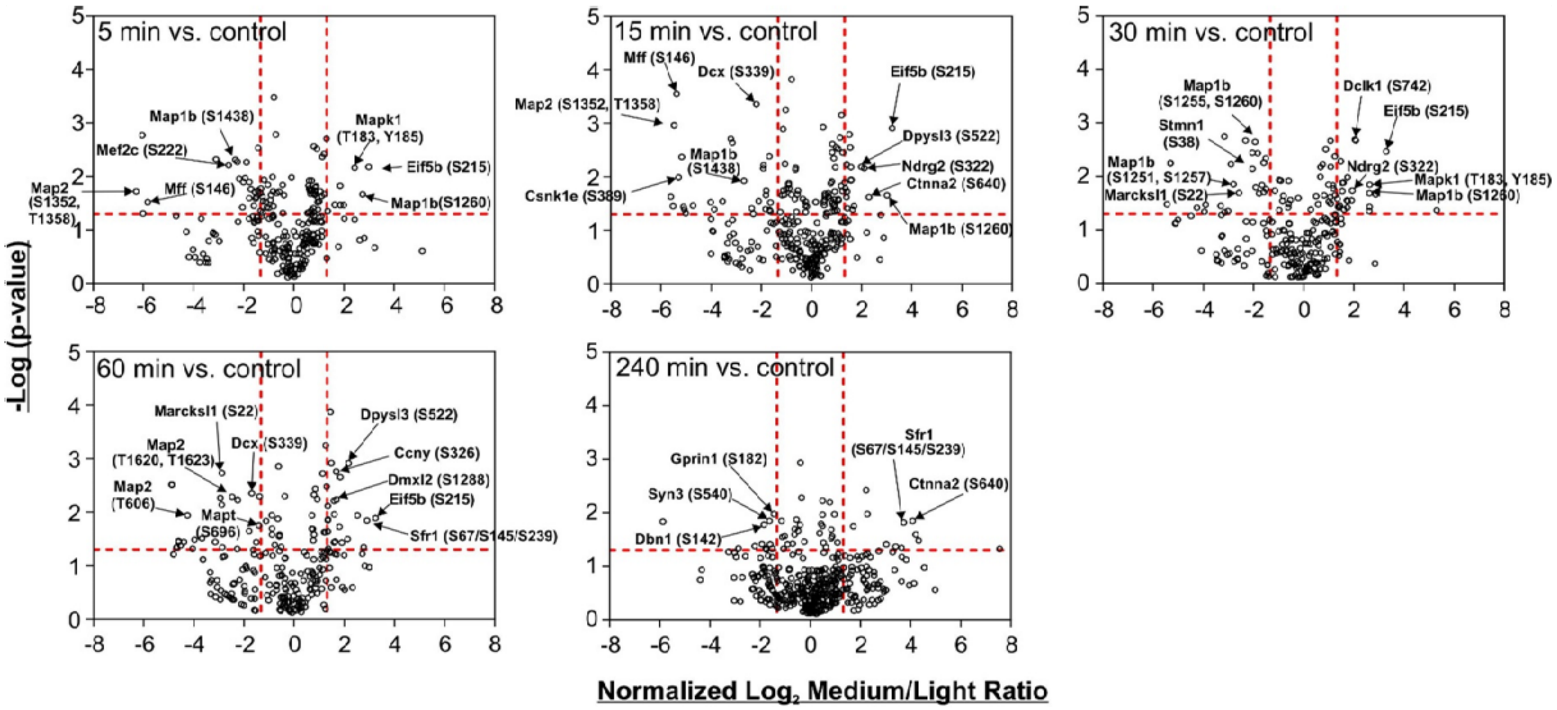

Figure S7. Volcano plots showing the temporal changes in abundance of identified phosphopeptides in neurons at different time points after glutamate treatment

Volcano plots depicting the quantified neuronal phosphoproteins with corresponding phosphopeptides identified at different time points following glutamate treatment. Selected neuronal proteins showing significant changes (±2.5-fold changes, $p \leq 0.05$) in phosphorylation are also highlighted.

Figure S8

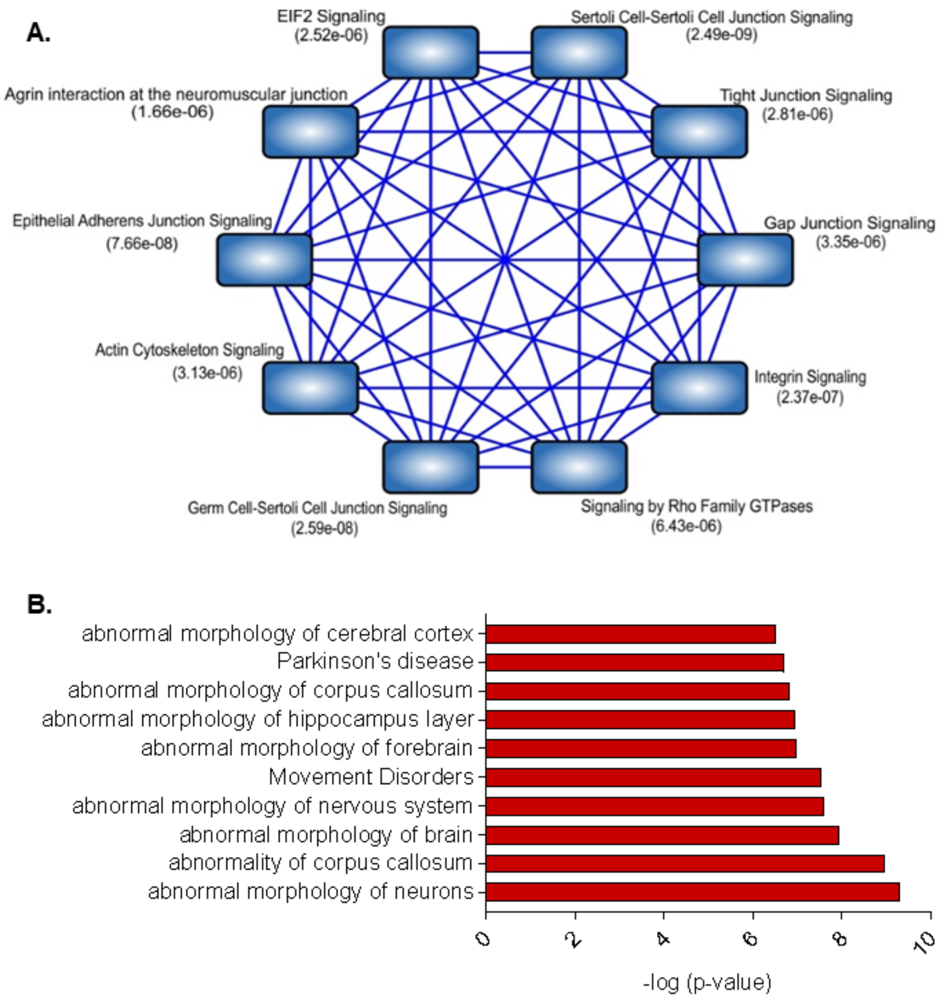

Figure S8. Top canonical pathways and disease and function annotations identified by IPA

A. Top 10 canonical pathways those are affected by the identified significantly perturbed neuronal proteins and phosphoproteins are presented. Pathways (overlapping pathways) containing identified sharing protein molecules are shown and *p*-values of individual pathways are in parentheses. B. Top 10 disease and function annotations identified by IPA are presented with corresponding *p*-values.

Figure S9

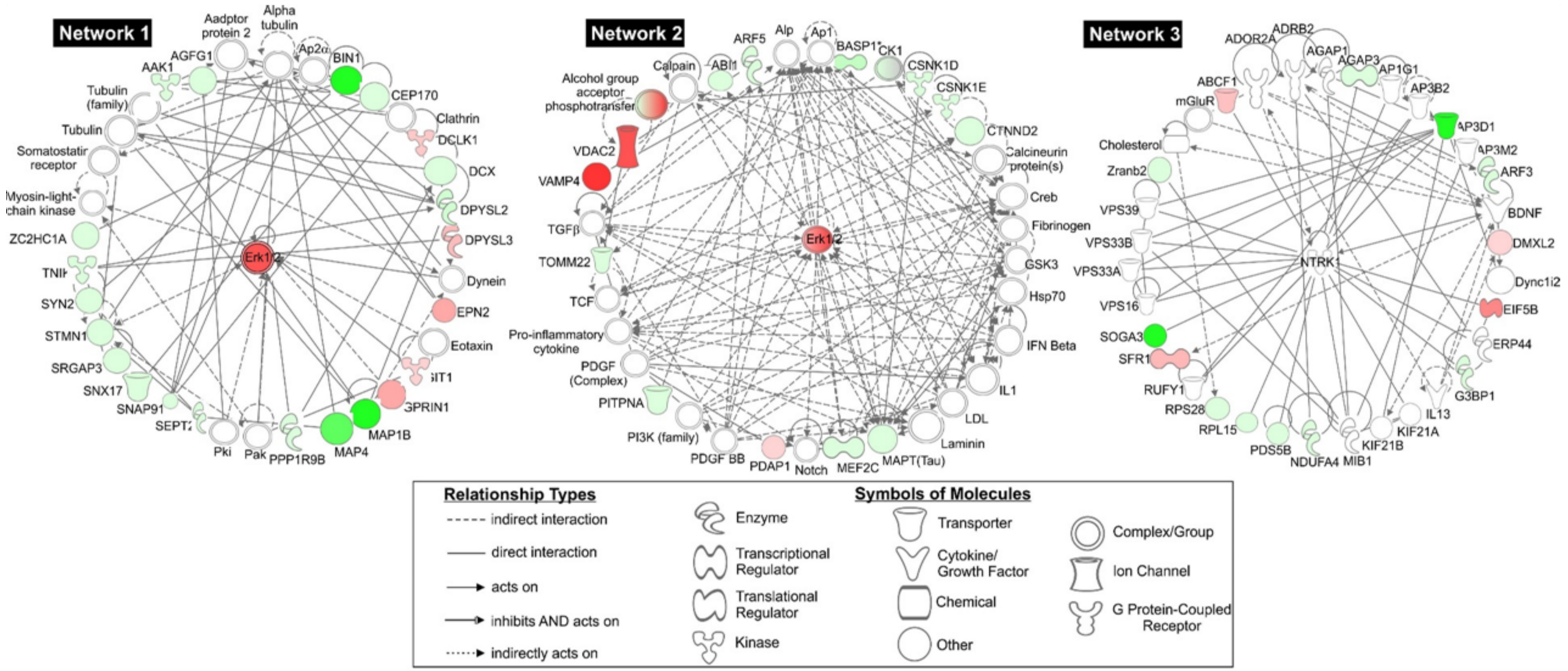

Figure S9. Top-ranked disease and function interaction networks identified by IPA

From the significantly changed neuronal proteins and phosphoproteins (±2.5-fold changes), IPA predicts the disease and function interaction networks. Three of the top-ranked networks are presented. The diseases and functions associated with the three networks and their scores are: Network 1, cell morphology, cellular assembly and organization, cellular development (score: 45); Network 2, Cellular Assembly and Organization, Embryonic Development, Organismal Development (score: 22); Network 3, molecular transport, protein trafficking, carbohydrate metabolism (score: 25). Protein molecules showing decrease in abundance or phosphorylation are highlighted in green in the networks, while neuronal proteins showing increased abundance or phosphorylation following glutamate treatment are presented in red symbols. . The score of each network is derived from a *p*-value that indicates the likelihood of the perturbed neuronal proteins in the network being found together due to random chance (Kramer, *et al. Bioinformatics* 2014, **30**(4):523-530).

Figure S10

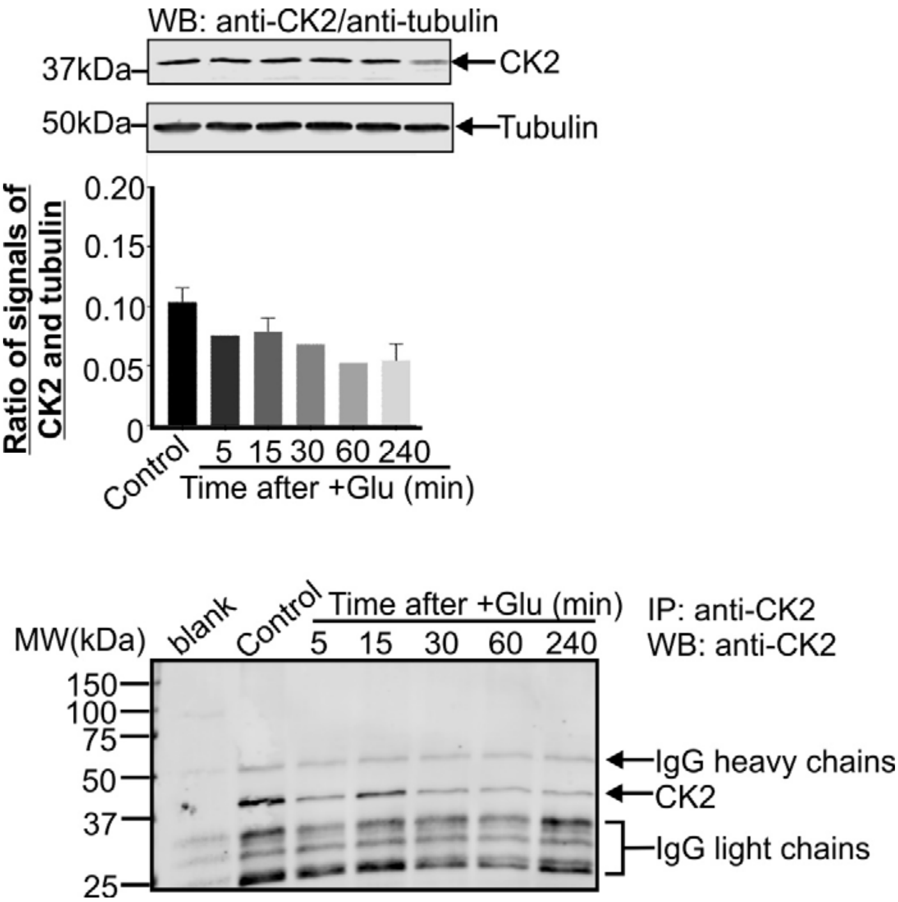

Figure S10. Western blot analysis of the CK2 expression in neurons and detection of CK2 immunoprecipitated from neuronal lysate

Lysates from control neurons and neurons over-stimulated with glutamate at 5, 15, 30, 60 and 240 min were monitored for expression levels of CK2 by Western blotting (top part of the upper panel). The expression levels of tubulin were used as the loading control. The ratios of densitometric values of the signals of CK2 versus those of tubulin are presented (lower part of the upper panel). CK2 in the neuronal lysates was purified by immunoprecipitation for kinase activity assay. The amounts of CK2 in the immunoprecipitates were monitored by Western blotting. The heavy and light chains of IgG in the immunoprecipitates are indicated. The portion of this blot showing the immunoreactive signals of CK2 was also shown in Figure 8B. The densitometric values of the immunoprecipitated CK2 were used to calculate the specific enzymatic activity of the immunoprecipitated CK2 in each neuronal lysate. Temporal changes of CK2 specific enzymatic activity in response to glutamate over-stimulation are presented in Figure 8B.

Supplemental References

1. Hossain, M. I. *et al.* A truncated fragment of Src protein kinase generated by calpain-mediated cleavage is a mediator of neuronal death in excitotoxicity. *J Biol Chem* **288**, 9696-9709, (2013).
2. Fricker, M., Tolkovsky, A. M., Borutaite, V., Coleman, M. & Brown, G. C. Neuronal Cell Death. *Physiol Rev* **98**, 813-880 (2018).
3. Grutter, M. G. Caspases: key players in programmed cell death. *Curr Opin Struct Biol* **10**, 649-655 (2000).
4. Lankiewicz, S. *et al.* Activation of calpain I converts excitotoxic neuron death into a caspase-independent cell death. *J Biol Chem* **275**, 17064-17071 (2000).
5. Wolf, B. B. *et al.* Calpain functions in a caspase-independent manner to promote apoptosis-like events during platelet activation. *Blood* **94**, 1683-1692 (1999).
6. McGinnis, K. M., Gnegy, M. E., Park, Y. H., Mukerjee, N. & Wang, K. K. Procaspase-3 and poly(ADP)ribose polymerase (PARP) are calpain substrates. *Biochem Biophys Res Commun* **263**, 94-99 (1999).
7. Wang, K. K. Calpain and caspase: can you tell the difference? *Trends Neurosci* **23**, 20-26 (2000).
8. Kowara, R., Chen, Q., Milliken, M. & Chakravarthy, B. Calpain-mediated truncation of dihydropyrimidinase-like 3 protein (DPYSL3) in response to NMDA and H2O2 toxicity. *J Neurochem* **95**, 466-474 (2005).
